# Relationship Between Maxillary Transverse Deficiency and Respiratory Problems: A Systematic Review of the Effectiveness of Devices over the Past Decade

**DOI:** 10.3390/jcm14248861

**Published:** 2025-12-15

**Authors:** Gaetano Ierardo, Fabiana Nicita, Iole Vozza, Antonella Polimeni, Valeria Luzzi

**Affiliations:** Department of Oral and Maxillo-Facial Sciences, “Sapienza” University of Rome, Via Caserta 6, 00161 Rome, Italy; gaetano.ierardo@uniroma1.it (G.I.); iole.vozza@uniroma1.it (I.V.); antonella.polimeni@uniroma1.it (A.P.); valeria.luzzi@uniroma1.it (V.L.)

**Keywords:** maxillary expansion, upper airway morphology, nasal airway patency, polysomnography, spirometry, sleep-disordered breathing, obstructive sleep apnea

## Abstract

**Background/Objectives**: Maxillary transverse deficiency is linked to impaired nasal breathing and pediatric sleep-disordered breathing. This systematic review evaluated the effects of maxillary expansion (ME) on upper-airway morphology and breathing function in growing patients. **Methods**: The search was conducted on PubMed/MEDLINE, Scopus, ScienceDirect, Cochrane CENTRAL, and gray literature (January 2015–April 2025). Eligible RCTs, controlled trials, and cohort/observational studies assessed airway morphology and/or respiratory outcomes after ME in pediatric/adolescent patients. Risk of bias was evaluated with RoB 2 (RCTs) and ROBINS-I (non-randomized studies). The findings were synthesized qualitatively and certainty graded with GRADE. **Results**: Forty-one studies were included. Imaging consistently showed enlargement of the nasal cavity and nasopharynx after expansion, whereas the effects in the oropharynx and hypopharynx, as well as in the maxillary sinuses, were smaller or variable. Objective patency improved in several studies (higher peak nasal inspiratory flow, reduced nasopharyngeal obstruction, and nasal resistance), whereas computational fluid dynamics generally showed non-significant trends toward lower resistance. Spirometry improved, particularly in oral breathers (gains in FEV_1_, FVC, FEF25–75%). Polysomnography indicated reductions in AHI and improved oxygenation in some pediatric OSA cohorts, although other RCTs reported null PSG effects. Caregiver-reported sleep and quality-of-life outcomes were consistently enhanced. Device design modestly influenced regional widening, but overall respiratory effects were similar across expanders. By GRADE, certainty was low for airway morphology and very low for breathing function. **Conclusions**: In growing patients, ME reliably enlarges upper-airway compartments, especially the nasal cavity and nasopharynx, yet functional improvements are heterogeneous. Standardized outcomes and integrated morphological–functional assessments are needed to strengthen the evidence base.

## 1. Introduction

Normal nasal breathing is essential for healthy craniofacial growth and overall development in children [1,2,3]. Impaired nasal airflow is frequently associated with maxillary constriction, a transverse skeletal deficiency of the upper jaw, which affects 2.7–23.3% of individuals under 18 years of age [4,5]. This condition often manifests as a high, narrow palate and a posterior crossbite, elevating the nasal floor and reducing nasal cavity volume [6,7]. Up to 47% of children with nasal breathing problems present maxillary constriction, highlighting a close relationship between craniofacial morphology and airway function [3]. The existence of a bidirectional association between nasal obstruction and maxillary development means that chronic mouth breathing can alter muscle balance and inhibit transverse maxillary growth, while a narrow maxilla further limits nasal airflow, perpetuating obstruction [8,9]. Long-term oral breathing is also linked to dentofacial changes, increased facial height, and altered tongue posture [10,11]. Moreover, reduced nasal patency contributes to sleep-disordered breathing (SDB), from primary snoring to obstructive sleep apnea (OSA) [12,13], which affects 1–5% of children and is associated with cognitive, behavioral, and cardiovascular complications [14]. Craniofacial factors such as a high-arched palate and maxillary constriction are recognized risk factors for upper-airway narrowing, even in the absence of adenotonsillar hypertrophy [15,16].

Maxillary expansion (ME) is an established orthopedic-orthodontic procedure for correcting transverse deficiency during growth. By separating the midpalatal suture, ME widens the maxilla and displaces the lateral nasal walls outward, increasing nasal cavity dimensions and potentially improving nasal airflow [17,18]. Several imaging studies have confirmed significant volumetric gains in the nasal cavity and nasopharyngeal space after ME, accompanied by reductions in nasal resistance and mouth-breathing patterns [19,20,21]. Nevertheless, the magnitude and persistence of these functional benefits remain debated, as some studies report transient improvements or interindividual variability depending on age, appliance type, and follow-up duration [22,23,24,25]. In recent years, several systematic reviews and meta-analyses have evaluated RME and other orthopedic or functional orthodontic interventions in children with SDB or OSA, primarily based on polysomnographic or oximetric outcomes, such as the apnea–hypopnea index (AHI) and oxygen saturation (SpO_2_) [26,27,28,29]. These reviews often include heterogeneous treatment modalities and mixed populations of pediatric and adult patients, and they rarely provide a unified synthesis of three-dimensional upper-airway imaging, objective nasal patency indices, and sleep-related respiratory parameters in growing subjects with transverse maxillary deficiency as the main orthodontic indication. 

Therefore, this systematic review aims to provide an updated and methodologically robust synthesis of the effects of ME therapy on upper-airway morphology, nasal resistance, and respiratory function in growing patients with transverse maxillary deficiency. Specifically, it focuses on pediatric and adolescent populations with growth potential, evaluates both structural (imaging-based) and functional outcomes, and assesses the consistency of these effects across different expansion protocols and study designs.

## 2. Materials and Methods

### 2.1. Registration and Protocol

This systematic review was created in accordance with the 2020 guidelines established by the Preferred Reporting Items for Systematic Reviews and Meta-Analyses (PRISMA) [30]. Before writing this paper, a comprehensive protocol detailing the methodology was created. The review has been recorded on the CRD York website, PROSPERO (CRD420251058335). 

### 2.2. Eligibility Criteria 

The research question was defined based on the PICOS (Population, Intervention, Comparison, Outcomes, Study design): (P) Pediatric and adolescent patients with maxillary transverse deficiency (typically 5–15 years; mixed/early permanent dentition), with or without mouth breathing, SDB/OSA; (I) Treatment with orthopedic or orthodontic maxillary expansion therapy, including any form of palatal expansion performed with fixed or hybrid appliances, regardless of the specific design or activation protocol; (C) A direct comparison between different types of expanders or treatment protocols was considered when available, otherwise, pre-treatment versus post-treatment comparisons within groups were analyzed; (O) Morphological and functional parameters related to airway and respiratory performance, such as changes in upper airway volume, nasal airflow, airway resistance, and respiratory indices (e.g., AHI, SpO_2_), as well as subjective or parent-reported improvements in breathing and quality of life; (S) Randomized controlled trials (RCTs), controlled clinical trials, cohort studies, and observational studies. Consequently, the following research question was posed: In growing patients with maxillary transverse deficiency, how does maxillary expansion therapy affect upper airway morphology and respiratory function, and to what extent are these effects consistent across different expansion protocols and study designs? The eligibility criteria for inclusion in this systematic review are outlined in Table 1.

### 2.3. Search Strategy

A comprehensive literature search was conducted using the following electronic databases: PubMed/MEDLINE, Scopus, ScienceDirect, and the Cochrane Library (CENTRAL). In addition, gray literature was explored through Google Scholar. The search was limited to articles published between January 2015 and April 2025. No restrictions were applied regarding language or country (studies in all languages will be considered, provided a complete English translation is available or achievable). The search strategy combined keywords, MeSH (Medical Subject Headings), and free-text terms related to maxillary transverse deficiency, respiratory function, and expansion devices. Boolean operators (“AND”, “OR”) were used to link the search terms (Table 2). 

### 2.4. Screening and Selection Process

All articles identified through a search across various databases were imported into reference management software (Mendeley Desktop, version 1.19.8), where duplicates were eliminated. Following this, two reviewers independently reviewed the titles and abstracts of the remaining studies to determine their relevance to the research question. Case reports, case series, reviews, and meta-analyses were excluded. Studies containing relevant search terms in the title and/or abstract were selected for full-text review. The two reviewers independently evaluated the full-text articles of potentially relevant studies based on predefined eligibility criteria. Studies that met all criteria were included in data extraction, while those that did not were excluded, with the reasons documented. Any disagreements between the two reviewers at this stage were discussed and resolved by consensus. If disagreements persisted, a third independent reviewer was consulted. 

### 2.5. Data Extraction

The two reviewers (GI and FN) independently extracted the data using a standardized template that included the following information: authors, year of publication, country, study design, treatment group, treatment type, control group, outcomes measured, follow-up duration, and conclusions. When discrepancies arose between data extractions, the third reviewer (VL) was consulted to resolve disagreements. A consensus approach was used to finalize the data, which was then compiled into a standardized database to ensure completeness and consistency. 

### 2.6. Data Synthesis

Given the substantial heterogeneity among the included studies, in terms of imaging modalities, expansion protocols, and respiratory outcome measures, a meta-analysis was not feasible for most variables. Therefore, the findings were primarily integrated through a structured qualitative synthesis.

### 2.7. Quality Assessment

Two reviewers (GI and FN) independently assessed the methodological quality and risk of bias of the included studies. Discrepancies were resolved through discussion or, if necessary, by consulting a third reviewer (VL). For randomized controlled trials (RCTs), the Cochrane Risk of Bias 2.0 (RoB 2.0) tool [31] was applied. This instrument evaluates potential sources of bias across five domains: (D1) randomization process, (D2) deviations from intended interventions, (D3) missing outcome data, (D4) measurement of the outcome, and (D5) selection of the reported result. Each domain was rated as having low risk of bias, some concerns, or high risk of bias, and an overall judgment will be made accordingly. For non-randomized studies, including prospective cohort studies, the ROBINS-I (Risk Of Bias In Non-randomized Studies—of Interventions) tool [32] was used. ROBINS-I assesses risk of bias across seven domains: (D1) bias due to confounding, (D2) classification of interventions, (D3) selection of participants into the study, (D4) deviations from intended interventions, (D5) missing data, (D6) measurement of outcomes, and (D7) selection of the reported result. Judgments were categorized as low, moderate, serious, critical risk of bias, or no information.

### 2.8. Certainty of Evidence Assessment

The certainty of evidence for each outcome (airway morphology and breathing function) was assessed using the Grading of Recommendations, Assessment, Development, and Evaluation (GRADE) framework [33]. Five primary domains were considered: risk of bias, inconsistency, indirectness, imprecision, and publication bias, and rated each as “not serious,” “serious,” or “very serious” based on pre-specified criteria. Risk of bias was assessed using RoB 2 for randomized trials and ROBINS-I for non-randomized studies and summarized at the outcome level using sample-size weighting. Inconsistency was evaluated by examining the variability in direction and magnitude of effects across studies and by considering methodological and clinical differences. Indirectness was judged against the review PICO, considering population characteristics, intervention protocols (including co-interventions), comparators, and the clinical relevance of endpoints. Imprecision was assessed based on the number of studies and participants, the presence or absence of pooled effect estimates, and confidence intervals. Publication bias was evaluated qualitatively, considering potential small-study effects, selective reporting, and gray literature. In addition, the effect direction was summarized to indicate whether the preponderance of evidence favored improvement, showed null/mixed results, or worsening. Finally, the overall certainty for each outcome was categorized as high, moderate, low, or very low [34].

## 3. Results

### 3.1. Article Selection

**Figure 1:**
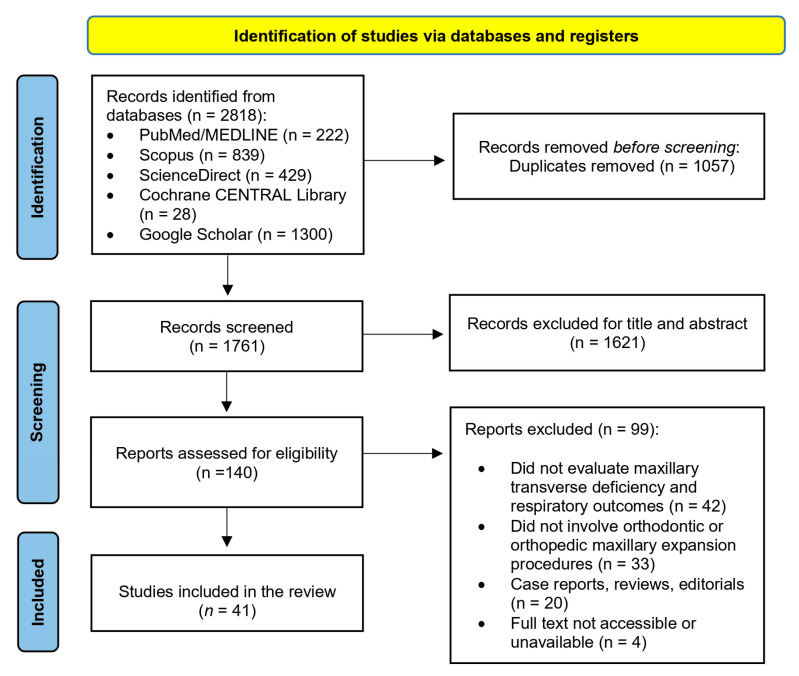
Selection of articles summarized using a PRISMA Flowchart.

The preliminary database search identified 2818 records across all sources. After the elimination of duplicates, 1761 unique articles remained. These records were subsequently screened based on their titles and abstracts, resulting in the exclusion of 1621 articles that were either irrelevant to the research topic or did not meet the predefined inclusion criteria. Following the initial screening, 140 articles were selected for full-text review. After a comprehensive assessment of the full texts, 99 articles were excluded for reasons including the lack of respiratory outcome measures, the absence of orthodontic expansion interventions, inappropriate study design, or unavailability of data. Ultimately, 41 studies met all eligibility criteria and were included in the qualitative synthesis. The study selection process is illustrated in Figure 1.

### 3.2. Characteristics of Included Studies

A total of forty-one studies published between 2015 and 2024 met the inclusion criteria and were included in this systematic review. The designs encompassed prospective longitudinal studies [35,36,37], randomized controlled trials (RCTs) [38,39,40,41,42,43,44,45,46,47], as well as prospective [48,49,50] and retrospective cohort/comparative studies [21,51,52,53,54,55,56,57,58,59,60,61], retrospective [62,63,64,65,66,67] and prospective controlled clinical trials [68] or non-controlled clinical studies [22,69,70,71,72], and prospective non-randomized studies [73].

The studies were conducted across Italy [35,36,39,41,48,53,55,57,60,61], Brazil [42,47,49,62,68,69,70,72], the USA [21,45,50,51,52,54,65], Turkey [44,46,56], Germany [40,51], UK [71], Canada [38], Australia [43,63], Denmark [63,65], Sweden [58], China [59], Korea [64], Spain [66], Saudi Arabia [67], Greece [22], Malaysia [37], India [73]. Sample sizes ranged from 11 to 91 participants, with mean ages most commonly between 5 and 15 years. Both male and female participants were represented, predominantly in mixed dentition stages.

Regarding the interventions, most studies evaluated tooth-borne RPE devices, including traditional Hyrax and Haas-type expanders [21,22,35,36,37,38,39,40,41,42,43,44,45,46,48,50,51,52,53,54,55,56,57,58,59,61,62,63,64,65,66,67,68,70,72,73] and modified or customized expanders [49,51,69,71]. The studies also analyzed hybrid-Hyrax/miniscrew-assisted expansion appliances [38,40,42,43,44,45,46,51,54]. A smaller subset compared different expansion protocols or devices, such as slow maxillary expansion with Leaf Expander [53], a keyless tooth-borne appliance with a Keles expander [43], fan-type (FE) and differential-opening (EDO) expanders [47], alternate RME and constriction (Alt-RAMEC) protocols [73], asymmetric RME (ARME) [56], and combined RME treatment with a Delaire facemask [60].

Three main methodological approaches to comparison were identified: controlled trials with untreated or growth-matched groups [38,41,62,63,64,65,66,67], interventional comparisons between different expansion appliances or protocols [38,39,40,42,43,44,45,46,47,53,55,56,60,73], and within-subject studies without external controls [22,35,36,37,42,43,45,47,48,49,50,51,54,56,57,58,59,60,61,68,69,70,71,72,73]. 

The analyzed outcomes were categorized into three main domains (airway morphology, respiratory function, and patient-reported/sleep-related outcomes). Airway morphology was assessed through cone-beam computed tomography (CBCT) for volumetric and dimensional airway analyses (nasal cavity, nasopharynx, oropharynx, hypopharynx) [21,22,35,36,37,39,42,43,45,46,47,48,49,51,52,53,54,55,56,57,58,59,60,61,62,63,64,65,66,67,68,69,70,71]. Respiratory and functional outcomes were assessed by nasal airway resistance, peak nasal inspiratory flow (PNIF), spirometric variables (FEV_1_, FVC, FEF25–75%, tidal volume, inspiratory time percentage), oxygen saturation (SpO_2_), and apnea–hypopnea index (AHI) [35,36,37,38,40,41,46,48,57,59,72]. Patient-reported and sleep-related outcomes, such as quality of life, parental perception of breathing and sleep quality, and behavioral changes, were recorded in some studies [50,69,72].

The follow-up periods ranged from immediate post-expansion evaluations to long-term assessments that extended up to 35 months. Short-term evaluations (immediate to 6 weeks) were performed in some studies [22,40,41,44,49,51,69,71]. Intermediate follow-ups (3–6 months) were the most frequent [22,37,38,40,43,44,46,47,48,49,52,54,56,57,58,59,62,67,68,70,72,73]. Medium- (7–12 months) and long-term (>1 year) follow-up were reported in ten [35,36,39,42,50,53,55,57,61,64] and six [21,45,60,63,65,66] studies, respectively. An overview of the studies included is presented in Table 3.

### 3.3. Airway Morphology

Across the 34 included studies, maxillary expansion consistently enlarged the nasal cavity and nasopharyngeal compartments, with more variable effects in the oropharynx/hypopharynx and maxillary sinuses. CBCT studies documented meaningful total–airway gains after RME (e.g., +3.656 mm^3^ at 12 months and +5.394 mm^3^ post-treatment), alongside skeletal widening of the palate (inter-palatal foramen distance increasing from 26.6 to 29.1 mm) [35,36]. A short-term study even showed large absolute volume expansion (+7.273 mm^3^; +11.5%) over 25 days, supporting a true treatment effect [51]. Studies with matched controls confirmed that natural growth contributes substantially; nevertheless, net gains attributable to expansion remained detectable (1353 mm^3^), as quantified via regression modeling [66]. Other matched cohorts reported within-group increases without clear inter-group superiority for total pharyngeal volume or minimal cross-sectional area (MCA) [63,65,67]. By contrast, a case–control CBCT study reported broader post-RME improvements across the nasopharyngeal, retropalatal, and retroglossal segments, including a significant advantage for retropalatal MCA [64]. In terms of temporal pattern, airway volume and MCA increased immediately post-expansion and were partly maintained during early retention: volume regressed modestly but remained above baseline, whereas MCA generally stayed elevated [22]. Similarly, acoustic rhinometry showed immediate anterior nasal improvement, with some recurrence after containment, alongside a more sustained increase in one nasal volume metric [49].

The strongest and most reproducible effects were observed in the nasal cavity and nasopharynx. In mouth-breathing children, RME widened both the anterior and posterior nasal floor by about 2.8 mm and increased the combined nasal–nasopharyngeal volume (+1646 mm^3^), while oropharyngeal change was not significant [69]. However, the study by Cappellette et al. [68] reported that mouth-breathers showed greater increases in nasomaxillary and oropharyngeal dimensions than controls. Landmark-based and volumetric assessments showed that both the rapid maxillary expansion (RME) and slow maxillary expansion (SME) techniques increased nasal widths and total nasal volume (RME: +1.68 cm^3^ and +3.13 mm; SME: +1.25 cm^3^ and +2.67 mm) [39]. Adolescents treated with either tooth-borne or bone-borne RME also exhibited significant increases in nasal cavity and nasopharyngeal volumes [54]. Over 12 months, percentage increases are substantial (~19–29% in the nasal cavity and ~25–63% in the nasopharynx) with significant gains in minimal cross-sectional area (CSmin) and a stronger nasopharyngeal/CSmin response in younger patients [61]. Additional studies reported increases in nasopharyngeal dimensions in patients with OSAS undergoing orthopedic protocols [60] and in children with OSA and maxillary restriction [37], whereas a small prospective series did not detect significant overall airway-volume changes [58]. In an age-matched comparison, nasal cavity volume rose significantly after RME, while pharyngeal volumes largely normalized rather than exceeded growth effects [65]. 

Effects in the oropharynx and hypopharynx were heterogeneous. The largest absolute volume gain sometimes occurred in the oropharynx, whereas the laryngopharynx often changed little [51]. Several studies found no significant inter-device or inter-group differences [54,67,73], yet one case–control analysis found retropalatal MCA improvements exceeding those in controls [64], while clinically meaningful oropharyngeal widening has been documented in Class III/OSAS management [60] and at 12 months post-RME in longitudinal cohorts [61].

Linear skeletal and soft-tissue nasal changes mirrored the volumetric pattern. Beyond the nasal-floor widening [69], growing patients showed increases in nasal height (+2.5 mm), nasal width (+2.9 mm), nasal area (+235 mm^2^), maxillary width (+3.6 mm), and intermolar width (+6.5 mm) after RME [70]. Multi-arm comparisons reported significant gains in nasal floor (≈+3.0 mm) and nasal wall widths (≈+2.4–2.7 mm) across appliance designs [55]. Soft-tissue changes tracked skeletal enlargement nearly proportionally while controls remained stable [62]. Both MARPE and conventional RPE increased anterior and posterior nasal widths and reduced the nasal septal deviation angle in the short term, with several dimensional improvements sustained in the long term; MARPE particularly maintained posterior nasal cavity widening, whereas RPE showed larger intermolar gains [45]. Immediate 3D assessments with bonded RME further confirmed dentoalveolar expansion and nasopharyngeal enlargement [71].

Sinus responses were smaller and study-dependent. Significant right/left maxillary sinus volume increases are reported in controlled mouth-breathing cohorts [68] and with both symmetric and asymmetric protocols [56], while both SME and RME produced comparable sinus enlargement in growing patients [53]. Conversely, several studies found limited or non-significant sinus changes [54,71,73], or increased inter-sinus distance without a corresponding increase in volume [37].

Device design and protocol generally had a modest influence on global airway enlargement, with level-specific exceptions. Hyrax, hybrid, and acrylic-cap expanders yielded comparable rhinologic effects despite age differences [51], and an RCT using acoustic rhinometry found similar MCA/volume improvements across tooth-, tooth-tissue-, and bone-borne appliances over three months [44]. In a multi-device CBCT study, total airway volume did not differ by expander, although nasopharyngeal change favored a Keles design over Hyrax, and pre-peak patients benefited more from hybrid anchorage [43]. Orthopedically, hybrid Hyrax produced greater superior widening than a conventional Hyrax (e.g., nasal cavity width +2.26 mm vs. +1.11 mm) [42]. An expander with differential opening achieved greater transverse gains in the lower third of the nasal cavity, especially posteriorly, than a fan-type device [47]. Faster activation (0.8 mm/day) produced greater increases in nasal and nasopharyngeal volume than a slower rate (0.5 mm/day), with few differences elsewhere [52]. RME and SME showed similar nasal and nasopharyngeal volumetric benefits and the expected reverse-V opening of the midpalatal suture [39,53]. Asymmetric protocols effectively targeted the affected side, whereas symmetric RME tended to provide larger overall increases in airway and sinus volumes [56]. Notably, some investigations highlighted high variability and a weak link between morphological enlargement and functional improvement [38].

Airway symmetry and morphology–function coupling deserve emphasis. In young children, RME reduced long-term left–right asymmetry in nasal cavity volumes and increased bilateral minimum cross-sectional widths [21]. Second, the correlation between volumetric gains and functional benefits was limited in some studies, even though caregiver-reported outcomes and focused 3D measures pointed to increased nasal area and symptomatic improvement [50].

### 3.4. Respiratory Function and Breathing Outcomes

#### 3.4.1. Polysomnographic and Respiratory Function Outcomes

Across prospective cohorts, RME was associated with meaningful improvements in oxygenation and sleep-disordered breathing in some studies. In two longitudinal series, peripheral oxygen saturation increased by ~5–6% and apnea–hypopnea index (AHI) decreased by ~3.6–4.2 events/h, with very large effect sizes for SpO_2_ and AHI (>4.0) and a smaller effect size for airway volume; notably, volumetric gains did not correlate with SpO_2_ or AHI changes [35,36]. In children with OSA and maxillary restriction, polysomnography (PSG) improved substantially following RME (AHI −5.43 events/h; ODI −5.76 events/h; lowest SpO_2_ +5.62%; arousal index −7.69 events/h; sleep efficiency +3.39%), while six children achieved AHI < 1 [37]. In contrast, a controlled trial reported no significant changes in AHI, ODI, or minimum SpO_2_ across different expander designs [46], and in children with persistent SDB after adenotonsillectomy, PSG trajectories did not differ between primary snoring and OSA groups despite symptomatic improvement [72]. 

Spirometry supported the functional benefits of RME, particularly among oral breathers. The study of Abate et al. [57] showed that spirometric indices such as Forced Vital Capacity (FVC), Forced Expiratory Volume in one second (FEV_1_), Tiffeneau index (FEV_1_/FVC), and mid-expiratory flow (FEF 25–75%) improved significantly in oral breathers, while nasal breathers showed improvements in FVC, FEF 25–75%, and tidal volume. The parameters approached those of nasal breathers by the 12-month follow-up [57]. 

#### 3.4.2. Nasal Airway Patency

Objective measures of patency generally improved after expansion. In the study by Di Vece et al. [48], endoscopic grading of nasopharyngeal obstruction significantly decreased (median choanal grade 1.5→1.0) with parallel reductions in total inspiratory and expiratory nasal resistance. The reduction in obstruction correlated positively with the decrease in expiratory resistance, suggesting that structural widening of the nasopharynx effectively translated into improved nasal airflow. These improvements were particularly relevant in patients whose initial obstruction was caused by adenoid hypertrophy, which was the most common finding before treatment.

Appliance comparisons suggested functional advantages for hybrid anchorage as tooth–bone–borne expanders were shown to produce higher post-expansion nasal airflow (+51–53 cm^3^/s) and lower resistance (−0.21 Pa·s/cm^3^) than tooth-borne devices, despite similar skeletal expansion [40]. 

The relationship between expansion and nasal respiratory function was further explored by Ottaviano et al. [41], who documented significant increases in Peak Nasal Inspiratory Flow (PNIF) and olfactory sensitivity (N-Butanol threshold) both immediately after expansion and at six months of follow-up. These findings demonstrate an improvement in objective nasal airflow and olfactory function in treated children compared with controls, who showed no spontaneous changes. However, anterior active rhinomanometry (AAR) values did not significantly change in either group, and self-reported nasal obstruction scores (SNOT-22) remained stable, suggesting that perceived nasal comfort does not always parallel objective airflow metrics.

A more detailed aerodynamic perspective was provided by Feng et al. [59], who used computational fluid dynamics to analyze upper airway airflow before and after expansion. Although the study did not find statistically significant changes in parameters such as pressure drop, midsagittal velocity, or wall shear stress, all indices showed a trend toward reduced airflow resistance, particularly during expiration. Patients with adenoidal hypertrophy maintained higher resistance values even after expansion, indicating that persistent soft-tissue obstruction may limit the functional benefits of skeletal widening.

In contrast, an AR/CBCT study across tooth-borne, bone-borne, and control groups found minimal and inconsistent changes in nasal airway dimensions and function [38]. The study also found no reliable correlation between functional and dimensional changes and noted that improvements, when present, were often asymmetrical. These findings highlight the variability in individual response and the importance of considering both skeletal and mucosal contributions to airway function.

#### 3.4.3. Subjective and Clinical Breathing Improvements

Reports from patients and caregivers consistently indicated better breathing and sleep after expansion. Mouth-breathing children reported broad symptomatic relief and better quality of life after RME, including reductions in nasal obstruction, snoring, daytime tiredness, restless sleep, and related complaints [69]. In a larger caregiver survey [50], the number of reported sleep apnea and breathing-related symptoms decreased sharply from a mean of 3.1 before treatment to 1.3 afterward. The most notable reductions were observed for snoring (both “snoring half the time” and “snoring loudly”), dry mouth upon waking, and heavy breathing during sleep, each of which improved by more than 60% to 80%. Symptoms of fatigue and poor sleep quality also improved, with parents reporting marked declines in daytime sleepiness, morning headaches, and difficulty waking. Behaviorally, children showed fewer attention and task-organization issues after expansion. Objective nasal cavity measurements confirmed a concurrent increase in nasal area, supporting the link between morphological and functional gains.

The findings from Bariani et al. [72] added further perspective by examining quality-of-life and behavioral outcomes in children with SDB following adenotonsillectomy. Scores on the Pediatric Sleep Questionnaire (PSQ) and the OSA-18 quality-of-life scale decreased significantly across all domains (snoring, drowsiness, and behavior) in both the primary snoring and OSA groups. Behavioral assessment using the Child Behavior Checklist revealed notable reductions in somatic complaints and aggressiveness, particularly in the OSA subgroup. Although cognitive functions remained largely stable, visuospatial working memory showed a near-significant improvement, suggesting subtle cognitive benefits accompanying better sleep and breathing.

### 3.5. Risk of Bias Evaluation

**Figure 2:**
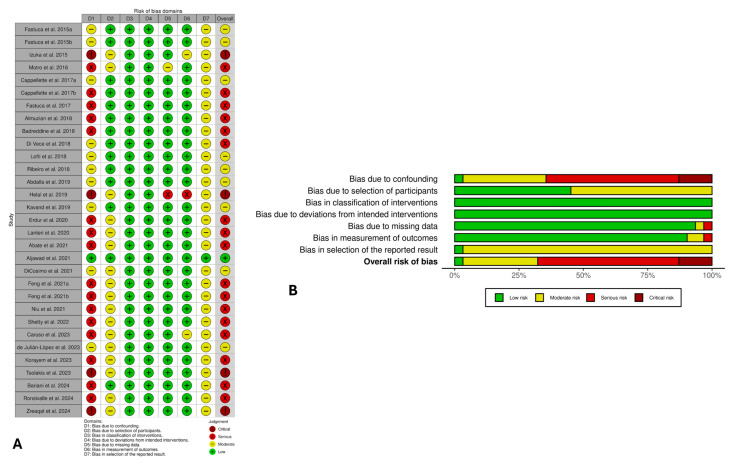
Risk of bias evaluation of non-randomized clinical trials with ROBINS-I: (**A**) The traffic light plot displays the domain-specific risk-of-bias judgments for each study [21,22,53,54,55,56,57,58,59,60,61,62,35,63,64,65,66,67,68,69,70,71,72,36,73,37,48,49,50,51,52], while (**B**) the weighted bar plot shows the overall distribution of risk-of-bias ratings across different domains.

The risk of bias for all non-randomized studies was assessed using the ROBINS-I tool (Figure 2). The overall methodological quality was limited, with many studies judged to be at serious risk of bias (n = 17), a few rated as critical (n = 4) or moderate (n = 9), and only one study rated as low risk. The domains of classification of interventions, deviations from intended interventions, missing data, and measurement of outcomes mainly showed low risk of bias, reflecting standardized RME protocols, clear intervention definitions, complete follow-up, and objective measurements. Conversely, the confounding and selection of participant domains were more frequently rated as moderate to serious risk, mainly due to the retrospective or uncontrolled design of several studies and the absence of statistical adjustment for growth or baseline airway variability. Selective reporting was rated as a moderate risk in several studies due to the absence of preregistered protocols.

**Figure 3:**
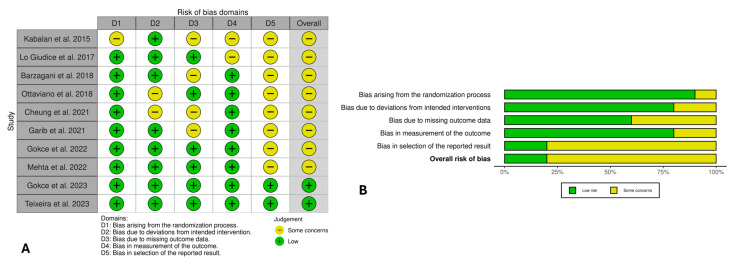
Risk of bias assessment of RCTs using RoB 2 with (**A**) traffic light plot for each study [38,39,40,41,42,43,44,45,46,47] and (**B**) the weighted bar plot.

The RoB 2 tool assessed the risk of bias for randomized controlled trials (Figure 3). Overall, most studies (n = 8) reported a “some concerns” rating, with only two studies reporting a low risk. The randomization process was generally well described, with adequate allocation concealment and balanced baseline characteristics. The domains of deviations from intended interventions, missing outcome data, and measurement of outcomes were predominantly rated as low risk, reflecting standardized expansion protocols, objective assessment methods, and complete follow-up. Conversely, the domain of selection of the reported result was most frequently associated with some concerns. 

### 3.6. Certainty of Evidence

For the two primary outcomes, certainty was “low” for airway morphology and “very low” for breathing function. Airway morphology remained low (mainly observational evidence with no serious downgrades), supported by a consistent direction of effect across structural sub-outcomes (nasal cavity and nasopharyngeal volumes/widths, minimal cross-sectional areas). Breathing function was rated very low, due to serious imprecision, inconsistency, and indirectness, reflecting heterogeneous sub-outcomes (PSG indices, nasal patency measures, and patient-reported outcomes) and fragmented sample sizes. Key limitations across outcomes included between-study heterogeneity, small to moderate samples, and the absence of pooled quantitative estimates. Details are reported in Table 4.

## 4. Discussion

This systematic review confirms that ME reliably enlarges the nasal cavity and nasopharynx, with changes appearing early and remaining partly above baseline during retention [22,35,36,51]. Growth is a confounding factor, as regression models indicate that a significant portion of enlargement reflects normal maturation, yet the residual growth-adjusted benefit (attributable to expansion) remains detectable, supporting that ME contributes an independent effect [66]. In contrast, effects in the oropharynx and hypopharynx are variable, with several studies showing segment-specific improvements (e.g., retropalatal), and matched comparisons do not demonstrate superiority overgrowth for global pharyngeal volume or minimal cross-sectional area [60,61,63,64,65,67,73]. This suggests that while ME occurs proximal to the midpalatine suture, significant skeletal changes occur near the nasal cavity/nasopharynx, whereas the more caudal segments are affected by soft tissue, tongue posture, and neuromuscular factors that have been variably characterized across studies [74,75].

Functional outcomes improve in many cases, but the link between structure and function is not strong. Prospective series report reductions in SDB indices and better oxygenation after RME, yet individual-level correlations between volumetric gains and PSG changes are weak or absent [35,36,37]. A randomized comparison across expander designs did not detect short-term improvements in PSG [46], and in children with persistent SDB after adenotonsillectomy, symptoms improved without a clear difference in PSG indices between subgroups [72]. Objective nasal airflow findings are more consistently positive: endoscopic obstruction grades and nasal resistance decrease after expansion [48], and peak nasal inspiratory flow and olfactory thresholds improve relative to controls [41]. Hybrid (tooth–bone–borne) anchorage shows higher airflow and lower resistance than tooth-borne devices despite similar skeletal widening, suggesting modest device-level functional differences in selected settings [40]. Conversely, multimodal assessments have also documented minimal or inconsistent changes in rhinomanometry and limited coupling between dimensional and functional metrics, and computational fluid dynamics studies generally show favorable trends that do not reach statistical significance, particularly when adenoidal hypertrophy persists [38,59]. Overall, soft-tissue burden appears to moderate the functional yield of skeletal widening.

Device and protocol choices primarily influence regional distribution, rather than the presence of the expansion effect itself. Across Hyrax, hybrid/MARPE, acrylic-cap designs, and even rapid versus slow protocols, broad nasal and nasopharyngeal benefits appear similar at a global level, with exceptions favoring superior/posterior nasal widening with hybrid anchorage or slightly larger posterior gains with faster activation in some reports [39,42,43,45,51,52,53]. Asymmetric strategies can address unilateral deficits, whereas symmetric expansion typically yields larger overall gains when the goal is bilateral improvement [56]. These findings support individualized selection based on the level and symmetry of obstruction, but the evidence base for device-specific superiority remains mixed.

Subjective outcomes reported by caregivers align more with the nasal signal than with PSG: many children are described as breathing and sleeping better after expansion, with reductions in snoring and daytime tiredness and improvements in behavior and quality of life [50,69]. These observations are clinically relevant but should be interpreted with care, given the predominance of unblinded designs and the limited use of prespecified minimal clinically important differences.

## 5. Limitations

Several methodological limitations of the available literature must be recognized and help explain the substantial heterogeneity of results. Most studies are observational, with modest sample sizes, heterogeneous or incompletely described control groups and growth modeling, differing imaging protocols, and functional endpoints ranging from PSG to PNIF, rhinomanometry, spirometry, and questionnaires, without consistent standardization. Clinical heterogeneity is also considerable, including variation in age, dentition stage, skeletal pattern, soft-tissue comorbidities, and concurrent therapies. Soft-tissue contributors are not consistently quantified or co-managed, which complicates attribution of functional change specifically to maxillary expansion and may blunt downstream effects. Within this framework, weak within-subject correlations between morphology and function are not unexpected, and the overall certainty of evidence, as judged with GRADE, is low for structural nasal/nasopharyngeal outcomes but very low for functional and sleep-related outcomes. Future research should prioritize standardized imaging protocols and longer retention follow-up, growth-aware comparators (matched or randomized), core outcome sets that include PSG with clinically meaningful thresholds, airflow metrics, and patient-reported measures, as well as phenotype-based analyses (e.g., predominant mouth-breathing, adenoidal hypertrophy, residual SDB post-adenotonsillectomy, skeletal class). Integration of computational fluid dynamics with systematic soft-tissue assessment may help clarify why similar volume changes yield different functional results. Well-powered RCTs comparing tooth-borne and hybrid anchorage with extended follow-up are especially needed to identify which subgroups derive the greatest and most durable clinical benefit.

## 6. Conclusions

Maxillary expansion is an effective orthopedic procedure for enlarging the upper airway, particularly the nasal cavity and nasopharynx, in growing patients. The intervention provides consistent skeletal widening, which can facilitate nasal breathing and reduce airway obstruction in selected cases. Across the different expansion devices assessed, broadly similar skeletal and airway effects were reported, and no consistent evidence emerged to support the superiority of any device over another for respiratory or sleep-related outcomes. However, functional improvements are not uniformly achieved, and polysomnographic results remain heterogeneous across studies. Benefits for respiratory function and sleep are most evident in children with significant transverse deficiency or SDB and are optimized when expansion is integrated with comprehensive clinical management and tailored patient selection. 

## Figures and Tables

**Table 1:** Inclusion and exclusion criteria.

Inclusion Criteria	Exclusion Criteria
**Population (P)**	
- Pediatric and adolescent patients (5–15 years) diagnosed with maxillary transverse deficiency	- Patients ≥ 16 years
- Mixed or early permanent dentition	- Syndromic or craniofacial malformation cases
- Mouth-breathing, nasal obstruction, or sleep-disordered breathing conditions	- Previous orthognathic or SARPE surgery
	- Concurrent airway surgery (e.g., adenoidectomy, tonsillectomy) during evaluation period
**Intervention (I)**	
- Orthopedic/orthodontic maxillary expansion (e.g., RME, RPE, MARPE, tooth-borne appliances)	- Expansion combined with surgical procedures was not analyzed separately
- Any activation protocol	- Bone distraction
**Comparison (C)**	
- Pre- post-expansion comparisons within subjects - Comparisons between different expansion designs or protocols - Comparisons with untreated/growth control groups	
**Outcomes (O)**	
- Changes in upper airway volume (assessed via CBCT, MRI, or other imaging techniques)	- Studies focusing solely on occlusal, skeletal, or aesthetic outcomes - Studies that do not evaluate respiratory outcomes
- Nasal airflow or nasal resistance (measured by rhinomanometry or other functional tests)	
- Respiratory function (AHI, SpO_2_, ODI, spirometry indices)	
- Self-reported improvements in breathing function using validated questionnaires	
**Study Design (S)**	
- Randomized controlled trials (RCTs)	- Case report
- Controlled clinical trials (CCTs)	- Case series
- Non-controlled clinical trials	- Narrative or systematic reviews
- Cohort studies	- Meta-analyses
- Observational studies	- Letters to the editor
	- Editorials
	- Book chapters
	- In vitro and animal studies
**Other**	
- Full-text, peer-reviewed studies published between 2015 and 2025	- Studies without an accessible full-text - Studies with irretrievable or incomplete data - Non-English papers without a reliable translation

**Table 2:** Database-Specific Search Strategies.

Database	Search Strategy
PubMed/MEDLINE	((“maxillary expansion”[Title/Abstract] OR “palatal expansion”[Title/Abstract] OR “maxillary constriction”[Title/Abstract] OR “narrow maxilla”[Title/Abstract] OR “transverse maxillary deficiency”[Title/Abstract]) AND (“airway”[Title/Abstract] OR “nasal breathing”[Title/Abstract] OR “airway obstruction”[Title/Abstract] OR “sleep apnea”[Title/Abstract] OR “airway volume”[Title/Abstract]) AND (“rapid maxillary expansion”[Title/Abstract] OR “rapid palatal expansion”[Title/Abstract] OR “RME”[Title/Abstract] OR “MARPE”[Title/Abstract] OR “miniscrew-assisted rapid palatal expansion”[Title/Abstract]))
Scopus	(TITLE-ABS-KEY (“maxillary expansion” OR “palatal expansion” OR “maxillary constriction” OR “narrow maxilla” OR “transverse maxillary deficiency”) AND ALL (“airway” OR “nasal breathing” OR “airway obstruction” OR “sleep apnea” OR “airway volume”) AND ALL (“rapid maxillary expansion” OR “rapid palatal expansion” OR “RME” OR “MARPE” OR “miniscrew-assisted rapid palatal expansion”))
ScienceDirect	(“maxillary expansion” OR “palatal expansion”) AND (“airway” OR “sleep apnea”) AND (“rapid maxillary expansion” OR “MARPE”)
Cochrane CENTRAL Library	(maxillary expansion OR palatal expansion OR transverse maxillary deficiency) AND (respiratory function OR airway obstruction OR nasal breathing OR sleep apnea) AND (rapid palatal expansion OR RPE OR MARPE OR RME)
Google Scholar	(“maxillary transverse deficiency” OR “maxillary constriction” OR “narrow maxilla” OR “posterior crossbite”) (“rapid palatal expansion” OR “RPE” OR “miniscrew-assisted rapid palatal expansion” OR “MARPE”) (“respiratory function” OR “airway volume” OR “nasal airflow” OR “sleep apnea”)

**Table 3:** Overview of the Characteristics of Selected Studies.

Study (Authors, Year)	Country	Study Design	Treatment Group	Treatment Type	Control Group	Outcomes Measured	Follow-Up Duration	Conclusions
Fastuca et al. (2015a) [35]	Italy	Prospective non-controlled longitudinal clinical study	22 M = 9 F = 13 Mean age: 8.3 ± 0.9	Haas-type maxillary expander	None	Airway volume (CBCT) Respiratory function metrics (spirometry, PSG)	12 months (post-expansion, after appliance removal)	RME produced significant increases in total airway volume and clinically meaningful improvements in oxygen saturation (SpO_2_) and AHI in growing patients. However, changes in airway volume did not correlate with changes in SpO_2_ or AHI, indicating that anatomical enlargement alone does not predict respiratory benefit.
Fastuca et al. (2015b) [36]	Italy	Prospective non-controlled longitudinal clinical study	15 M = 4 F = 11 Mean age: 7.5 ± 0.3	Haas-type maxillary expander	None	Airway volume (CBCT) Respiratory function metrics (spirometry, PSG)	12 months (post-expansion, after appliance removal)	RME produced significant morphological gains, increasing volumes of the upper, middle, and lower airway compartments. Patients with smaller baseline middle- and lower-airway volumes showed the greatest post-RME improvement in oxygen saturation (SpO_2_), indicating that baseline compartment size is a useful predictor of oxygenation benefit. Although AHI decreased overall, its change did not correlate with baseline airway dimensions, and morphology predicts SpO_2_ gains better than AHI response.
Izuka et al. (2015) [69]	Brazil	Prospective non-controlled clinical trial	25 M = 11 F = 14 Mean age: 10.5 ± 2.2	Modified Biederman-type appliance	None	Upper airway dimensions (CBCT): - Transverse width of the nasal floor; - Nasopharynx and nasal cavities volume; - Oropharynx volume. Quality of Life Questionnaire	Immediate (≈3 weeks post-expansion)	RME is an effective treatment that not only produces significant dimensional changes in the upper airway, specifically in the nasal cavity and nasopharynx, but also markedly improves the overall quality of life for patients with mouth breathing and maxillary atresia.
Kabalan et al. (2015) [38]	Canada	RCT	**Based on the RME appliance type:** 1. *Tooth-Borne (TB) Group:* 20 (5 M, 15 F) with a mean age of 14.1 years. 2. *Bone-Borne (BB) Group:* 20 (8 M, 13 F) with a mean age of 14.2 years.	TB group: Hyrax tooth-borne appliance BB group: bone-integrated miniscrew implant bone- anchored maxillary expansion device	21 M = 6 F = 15 Mean age: 12.9 ± unclear	Nasal airway dimensions (acoustic rhinometry and CBCT): - Minimum cross-sectional area; - Airway volume.	6 months (after appliance removal)	RME (tooth-borne or bone-borne) did not yield consistent, clinically meaningful improvements in nasal airway dimensions or function. Changes in nasal airway volume and function were minor, variable, and sometimes negative, indicating that any observed improvements were likely coincidental rather than treatment-related. RME should not be used with the primary goal of improving nasal airway function, as its effects are inconsistent and lack long-term predictability.
Motro et al. (2016) [51]	USA Germany	Retrospective multicenter comparative study	31 M = 12 F = 19 Mean age: 14.63 ± 0.38 **Based on the RME appliance type:** 1. *Hyrax Group:* 5 (gender not specified) with a mean age of 13.80 ± 0.12 years. 2. *Acrylic Cap Group:* 20 (gender not specified) with a mean age of 14.05 ± 0.31 years. 3. *Hybrid Group:* 6 (gender not specified) with a mean age of 17.25 ± 1.26 years.	1. Hyrax-type 2. Acrylic Cap-type 3. Hybrid-type (combination of molar bands with a skeletal anchorage unit)	None	Airway volume (CBCT): - Total airway volume; - Nasopharynx; - Oropharynx; - Laryngopharynx.	Immediate (≈25 days post-expansion)	RME produced significant enlargement of nasopharyngeal and oropharyngeal volumes, confirming a robust upper-airway response to expansion. No meaningful change in the laryngopharyngeal airway was detected, indicating the effect is primarily confined to superior airway levels. Therapeutic gains were comparable across appliance types and ages; Hybrid RME achieved airway improvements similar to those of tooth-borne designs, despite being used in older patients.
Cappellette et al. (2017a) [68]	Brazil	Prospective controlled clinical study	23 M = 12 F = 11 Mean age: 9.6 ± 2.3	Hyrax-type rapid expander	15 M = 9 F = 6 Mean age: 10.5 ± 1.9	Airway volume (CT): - Total nasomaxillary complex; - Nasal cavity; - Oropharynx.	3 months (post-retention)	RME produced a significant 3D volumetric enlargement of the nasomaxillary complex in mouth-breathing patients with transverse maxillary deficiency. Structure-specific gains were documented in the nasal cavity, oropharynx, and both maxillary sinuses, indicating a widespread morphologic response. RME is an effective intervention to increase airway-related volumes in this population, while functional benefits should be assessed separately.
Cappellette et al. (2017b) [70]	Brazil	Prospective non-controlled clinical study	61 M = 35 F = 26 Mean age: 9.6 ± unclear	Modified Hyrax expander, tooth-anchored	None	Skeletal and nasal dimension changes (PA cephalometric radiographs): - Nasal width; - Nasal height; - Nasal area.	3 months (post-RME)	RME produced significant increases in all transverse linear measurements of the maxilla and nasal cavity, alongside greater nasal cavity volume, supporting improved airflow and favorable craniofacial growth. RME was equally effective in boys and girls, and maxillary constriction showed no association with sex.
Fastuca et al. (2017) [55]	Italy	Retrospective comparative study	44 M = 20 (mean age: 8 years and 8 months ± 1 years and 2 months) F = 24 (mean age: 8 years and 2 months ± 1 years and 4 months) **Based on the RME appliance type:** 1. *HX-6 Group*: 15 (gender not specified), mean age not specified. 2. *HX-E Group*: 14 (gender not specified), mean age not specified. 3. *HS-E Group*: 15 (gender not specified), mean age not specified.	1. HX-6 Group: Hyrax expander anchored to permanent teeth; 2. HX-E Group: Modified Hyrax expander anchored to deciduous teeth; 3. HS-E Group: Modified Haas-type expander anchored to deciduous teeth.	None	Nasal cavity dimensions (CBCT): - Nasal floor width; - Nasal wall width.	7 months (post-retention)	The study investigates the effects of RME with different appliance designs and anchorage methods. RME effectively produces a significant skeletal transverse expansion of the nasal region in growing patients. The study found that no significant differences in nasal effects are expected whether the RME appliance is anchored onto deciduous teeth, with or without palatal acrylic coverage. This suggests that the choice between permanent or deciduous tooth anchorage, or the specific design of the expander, does not result in a statistically significant difference in terms of nasal expansion.
Lo Giudice et al. (2017) [39]	Italy	RCT	**Based on the type of maxillary expansion:** 1. *RME group*: 10 (6 M, 4 F) with a mean age of 10.4 ± 1.72 years. 2. *SME group*: 10 (4 M, 6 F) with a mean age of 10.5 ± 1.41 years.	Hyrax-type expander	None	Nasal cavity dimensions (CBCT): - Nasal cavity width; - Total nasal volume.	7 months (post-retention)	Both RME and SME effectively increased the dimensions of the nasal cavity. Skeletal expansion relative to dental expansion is a better indicator of protocol efficacy than absolute NW values.
Almuzian et al. (2018) [71]	UK	Prospective non-controlled clinical study	17 M = 8 F = 9 Mean age: 12.6 ± 1.8	A cast-cap appliance that incorporated a Hyrax screw	None	Volumetric changes of the upper nasopharyngeal airway spaces (CBCT): - Lower nasal cavity; - Upper nasopharynx; - Retropalatal (velo-pharyngeal) space.	Immediate (≈23 days post-expansion)	RME was found to be an effective dentoalveolar expander in growing patients. Upper nasopharynx volume increases after RME, while the upper retropalatal space decreases. Airway change follows a mushroom-like pattern with superior enlargement and midlevel narrowing.
Badreddine et al. (2018) [62]	Brazil	Retrospective controlled study	39 M = 23 F = 16 Mean age: 9.7 ± 2.28	Hyrax-type rapid expander	16 M = 9 F = 7 Mean age: 8.8 ± 2.17	Skeletal and soft tissue variables of the nasal cavity (CBCT)	3 months (post-retention)	RME produces significant short-term changes in nasal skeletal and soft-tissue structures in mouth-breathing children. Soft-tissue response closely mirrors skeletal expansion, with approximately 0.95 mm of soft tissue per 1 mm of skeletal increase. Findings support incorporating nasal soft-tissue effects into aesthetic planning for the nasal tip and base.
Barzagani et al. (2018) [40]	Germany	RCT	**Based on the RME appliance type:** 1. *TB group:* 19 (11 M, 8 F) with a mean age of 9.7 ± 1.5 years. 2. *TBB group:* 21 (10 M, 11 F) with a mean age of 10.2 ± 1.4 years.	TB group: conventional tooth-borne appliance TBB group: bone-integrated miniscrew implant bone- anchored maxillary expansion device	None	Nasal airflow and resistance (active anterior rhinomanometry, AAR)	Immediate (post-expansion)	Tooth–bone–borne RME produced higher nasal airflow and lower nasal resistance than tooth-borne RME. Findings support TBB RME when maxillary constriction coexists with upper airway obstruction. Expected benefit focuses on nasal breathing performance rather than appliance-specific skeletal expansion.
Di Vece et al. (2018) [48]	Italy	Prospective pilot study	30 M = 12 F = 18 Mean age: 8.7 ± 0.9	Hyrax-type rapid expander	None	Nasopharynx obstruction (rhinofibroscopy) Nasal airway resistance (rhinomanometry)	6 months (post-retention, after appliance removal)	RME reduces nasal resistance and the degree of nasopharyngeal obstruction, indicating a measurable physiological benefit for the upper airways. Upper airway patency improves after expansion, with the greatest value in patients with mild to moderate nasal obstruction. RME is adjunctive rather than a replacement for indicated medical therapy or adenoidectomy.
Lotfi et al. (2018) [52]	USA	Retrospective study	**Based on the RME activation rates:** 1. *Group A* (Higher Activation Rate, 0.8 mm/day activation): 20 (8 M, 12 F) with a mean age of 12.3 ± 1.9 years. 2. *Group B* (Slower Activation Rate, 0.5 mm/day activation): 20 (10 M, 10 F) with a mean age of 13.8 ± 1.3 years.	Hyrax-type rapid expander	None	Airway volume (CBCT): - Nasal cavity; - Nasopharynx; - Oropharynx; - Hypopharynx.	3 months (post-retention)	Airway volume change after RME is rate-dependent, with faster activation producing larger gains. Nasal cavity and nasopharynx volumes increase more with rapid activation than with slower protocols. Findings support modulating activation speed when prioritizing upper-airway enlargement in constricted patients.
Ottaviano et al. (2018) [41]	Italy	RCT	11 Gender: not specified Mean age: 8.27 ± 1.62	Hyrax-type rapid expander	11 Gender: not specified Mean age: 8.27 ± 1.25	Nasal respiratory function: - Nasal flow and patency (Peak Nasal Inspiratory Flow, PNIF); - Nasal resistance (AAR).	Immediate post-expansion or 1 month after enrolment (for control group); 6 months	RME enhances nasal physiology, with higher PNIF immediately and at six months, and improved N-butanol olfactory threshold. The likely mechanism is increased nasal airflow rather than measurable changes in standard resistance metrics. No significant change in nasal resistance on AAR. Clinically useful for managing crossbite in growing children while conferring ancillary benefits in airflow and smell perception.
Ribeiro et al. (2018) [49]	Brazil	Prospective observational study	19 M = 10 F = 9 Mean age: 8.9 ± unclear	Modified Biederman dento-supported expander type	None	Nasal cavity geometry (CBCT): - Minimum cross-sectional areas; - Nasal space volumes. - Nasal cavity patency (AR)	1 week after expansion; 6 months	Immediate enlargement of the anterior nasal cavity after RME, with regression toward baseline after the containment period. Nasal dimensional gains are short-lived and mainly anterior, with limited long-term impact on cross-sectional area. Primary indication remains correction of dentoalveolar problems rather than respiratory therapy.
Abdalla et al. (2019) [63]	Denmark Australia	Retrospective controlled clinical study	26 M = 12 F = 14 Mean age: 12.3 ± 2.3	TB Hyrax-type expander	26 M = 12 F = 14 Mean age: 12.33 ± 2	Upper airway dimensions (CBCT): - Minimal cross-sectional area; - Pharyngeal airway volume.	≈35 months (matched with controls)	Tooth-borne RME did not yield a significant increase in upper pharyngeal airway volume or minimal cross-sectional area versus controls, despite clear gains in intermolar and maxillary widths. Skeletal age was a significant predictor of airway change, with younger skeletal age showing more favorable airway responses. Dental and skeletal widening does not necessarily translate to upper-airway enlargement.
Helal et al. (2019) [50]	USA	Prospective observational study	91 M = 49 F = 42 Mean age: 7.6 ± 5	bonded Hyrax expander	None	Parental perceptions (16-item questionnaire) Nasal cavity dimensions (CBCT)	12 months (including 6-month interim assessment)	Parents reported better behavior, less daytime fatigue, and improved sleep and breathing after RME. Perceived gains were most evident for behavior and fatigue, with smaller but present improvements in sleep quality and breathing. CBCT models confirmed increased nasal cavity size, supporting a structural basis for breathing improvements.
Kavand et al. (2019) [54]	USA	Retrospective comparative study	**Based on the RME appliance type:** 1. *Tooth-Borne (TB) Group:* 18 (8 M, 10 F) with a mean age of 14.4 ± 1.3 years. 2. *Borne-borne (BB) Group:* 18 (6 M, 12 F) with a mean age of 14.7 ± 1.4 years.	TB group: Hyrax-type expander BB group: two palatal miniscrews connected to a jackscrew	None	Upper airway volume (CBCT): - Nasal cavity; - Nasopharynx; - Oropharynx; - Maxillary Sinus.	3 months (post-retention)	Both tooth-borne and bone-borne RME increased nasal cavity and nasopharynx volumes in adolescents. No meaningful intergroup difference in airway volume change was detected. Bone-borne RME may be preferred when aiming to minimize dentoalveolar side effects while achieving similar airway gains.
Erdur et al. (2020) [56]	Turkey	Retrospective comparative study	**Based on the RME type:** *1. Symmetric RME Group*: 30 (16 M, 14 F) with a mean age of 14.04 years. *2. Asymmetric (ARME) Group*: 30 (14 M, 16 F) with a mean age of 13.75 years.	RME group: Hyrax-type rapid expander ARME group: modified acrylic bonded appliances with an occlusal-lock mechanism on the unaffected side and with a Hyrax screw	None	Upper airway dimensions (CBCT): - Pharyngeal airway volumes; - Maxillary sinus volume.	3 months (post-retention)	Both symmetric RME and asymmetric ARME increased pharyngeal airway and maxillary sinus volumes. RME was effective for patients with bilateral maxillary deficiency. ARME effectively corrected true unilateral posterior crossbite and also increased airway and sinus volumes. Both protocols achieved meaningful orthopedic widening with favorable volumetric airway changes.
Lanteri et al. (2020) [53]	Italy	Retrospective case–control CBCT study	**Based on the maxillary expansion appliance type:** 1. *Slow Maxillary Expansion (SME) Group:* 22 (9 M, 13 F) with a mean age of 8.2 ± 0.6 years. 2. *Rapid Maxillary Expansion (RME) Group:* 22 (11 M, 11 F) with a mean age of 8.1 ± 0.7 years.	SME group: Leaf Expander RME group: Hyrax expander	None	Upper airway dimensions (CBCT): - Nasal cavity; - Nasopharynx; - Maxillary sinus.	≈11 months (range 10–14 months, post-retention)	Slow maxillary expansion (SME) is effective for treating maxillary hypoplasia in mixed dentition. SME increases pharyngeal airway volume and maxillary sinus volumes. SME and conventional Hyrax-type RME show comparable airway and sinus gains, with no significant differences. Both SME and RME enlarge the upper-airway and maxillary sinus volumes in growing patients.
Abate et al. (2021) [57]	Italy	Retrospective case–control clinical study	**Based on the baseline breathing patterns:** 1. *Oral Breathes*: 25 (12 M, 13 F) with a mean age of 15.2 ± 1.3 years. 2. *Nasal Breathers*: 25 (11 M, 14 F) with a mean age of 14.9 ± 1.7 years.	Hyrax-type rapid expander	None	Nasal respiratory function (spirometry): - Forced vital capacity; - Forced expiratory volume in the first second; - Tiffenau index; - Forced expiratory flow at 25–75% of vital capacity; - Tidal volume.	6 months after RME; 12 months after RME	RME improved breathing function in both oral and nasal breathers. Oral breathers showed greater gains, with significant increases in FEV1 and the Tiffenau Index, likely due to reduced peripheral airway resistance. In oral breathers, FVC, FEF25–75%, and tidal volume increased to values comparable to those of nasal breathers. Improvements were largely sustained, with no between-group differences in spirometric indices at 12 months.
Aljawad et al. (2021) [64]	Korea	Retrospective controlled clinical study	17 M = 3 F = 14 Mean age: 12.6 ± 1.8	Hyrax-type rapid expander	17 M = 4 F = 13 Mean age: 12.3 ± 1.5	Upper airway volume (CBCT): - Nasopharynx; - Oropharynx; - Minimal cross-sectional area.	≈10.5 months (post-retention)	RME produced significant increases in upper airway dimensions in the treated group, while matched controls showed no meaningful change. Overall post-treatment gains in airway volumes and cross-sections were greater with RME than in controls. The oropharyngeal segment improved, with a statistically significant rise in minimum cross-sectional area, most clearly at the retropalatal level. Findings support RME as an effective intervention for enlarging upper airway dimensions beyond natural growth.
Cheung et al. (2021) [43]	Australia	RCT	**Based on the RME appliance type:** 1. *Hyrax Group:* 19 (10 M, 9 F) with a mean age of 13.8 years. 2. *Hybrid-Hyrax Group:* 19 (8 M, 11 F) with a mean age of 14.3 years. 3. *Keles Group:* 13 (2 M, 11 F) with a mean age of 14.6 years.	Hyrax Group: conventional Hyrax expander Hybrid-Hyrax Group: tooth-bone-borne device Keles Group: Keles keyless expander	None	Upper airway dimensions (CBCT): - Total airway volume; - Nasal cavity volume; - Nasopharynx volume; - Oropharynx volume; - Hypopharynx volume.	6 months (post-retention)	RME produced only small increases in total upper airway volume across devices, ranging from 3.8% with Hyrax to 8.3% with Hybrid-Hyrax and 4.5% with Keles. No significant inter-appliance differences were detected for overall volume or most airway compartments. The nasopharynx was the only compartment showing a significant intergroup difference. Patients with smaller baseline airway volumes exhibited greater post-expansion gains. In pre-peak growth patients, Hybrid-Hyrax achieved significantly larger total airway volume increases than conventional Hyrax
DiCosimo et al. (2021) [21]	USA	Retrospective controlled observational study	28 M = 11 F = 17 Mean age: 9.86 ± 2.43	Hyrax-type rapid expander	20 M = 9 F = 11 Mean age: 10.41 ± 1.60	Upper airway dimensions (CBCT): - Nasal cavity volumes; - Nasopharyngeal volume; - Oropharyngeal volume; - Minimum cross-sectional widths.	24 months (post-RME)	RME produces significant and lasting improvements in upper airway dimensions. Patients showed a clear increase in nasal volume, minimum cross-sectional width, and nasopharyngeal volume compared with the control group. RME also contributed to improved nasal cavity symmetry by reducing the volume difference between the right and left nasal sides over time.
Feng et al. (2021a) [58]	Sweden	Comparative retrospective study	17 M = 11 F = 6 Mean age: 12.2 ± 1.3 **Based on adenoidal nasopharyngeal (AN) ratio**: 1. *Group 1 (AN ratio < 0.6, normal adenoids):* 10 with a mean age of 11.9 ±1.29 years. 2. *Group 2 (AN ratio ≥ 0.6, enlarged adenoids)*: 7 with a mean age of 12.6 ± 1.27 years.	Hyrax-type rapid expander	None	Upper airway morphology (CBCT): - Nasopharynx; - Retropalatal; - Retroglossal.	5.7 months (post-retention)	RME showed only a modest, non-significant tendency to enlarge the nasopharyngeal airway, with the signal more apparent in children with adenoid hypertrophy. The AN ratio trended downward (less obstruction) but did not reach statistical significance. Upper-airway dimensional changes were small and inconsistent, suggesting limited morphological impact in the short term. RME may have a modest morphological influence on AH patients, but the effects were not statistically robust and should not be relied upon to reduce nasopharyngeal obstruction.
Feng et al. (2021b) [59]	China	Cohort retrospective	17 M = 11 F = 6 Mean age: 12.2 ± 1.3 **Based on the AN ratio**: 1. *Group 1* (AN ratio < 0.6); 2. *Group 2* (AN ratio ≥ 0.6).	Hyrax-type rapid expander	None	Aerodynamic characteristics of the upper airway	≈5.2 ± 1.7 months (including active expansion + retention)	No significant change in airflow dynamics after RME, with CFD metrics (pressure drop, peak velocity, wall shear stress) remaining statistically unchanged. Any numerical reductions observed did not reach significance, suggesting short-term morphologic widening may not translate into measurable aerodynamic improvement.
Garib et al. (2021) [42]	Brazil	RCT	**Based on the RME appliance type:** 1. *HH Group:* 18 (10 M, 8 F) with a mean age of 10.80 ± 1.04 years. 2. *(CH) Group:* 14 (8 M, 6 F) with a mean age of 11.44 ± 1.26 years.	HH Group: Hybrid-Hyrax tooth-bone-borne device CH Group: Conventional Hyrax expander	None	Nasal airway dimensions (CBCT): - Nasal cavity width.	~11.38 months (HH group) and ~11.00 months (CH group)	Hybrid Hyrax achieved greater orthopedic widening at superior levels, with larger increases in nasal cavity and maxillary widths.
Niu et al. (2021) [65]	Denmark USA	Retrospective controlled clinical study	39 Gender: not specified Mean age: 10.40 ± 1.74	Hyrax-type rapid expander	29 Gender: not specified Mean age: 11.07 ± 1.45	Upper airway dimensions (CBCT): - Nasal cavity volume; - Pharyngeal airway total volume; - Partial PA volumes; - Minimal Cross-Sectional Area.	≈22.6 months (matched with controls)	RME significantly increases nasal cavity volume, with effects most evident when palatal width expansion exceeds 2 mm. Pharyngeal airway volume shows no net advantage over controls; within-group increases mainly reflect normalization rather than true surplus gain. Minimal cross-sectional area and minimal hydraulic diameter, initially reduced in RME patients, normalize to control levels after treatment.
Gokce et al. (2022) [44]	Turkey	RCT	46 M = 16 F = 30 Mean age: 12.8 ± 1.2 **Based on the RME appliance type:** 1. *Tooth Tissue-Borne (TTB) Group:* 15 (3 M, 12 F) with a mean age of 12.5 years. 2. *Tooth-Borne (TB) Group:* 15 (9 M, 6 F) with a mean age of 12.8 years. 3. *Bone-Borne (BB) Group:* 16 (3 M, 13 F) with a mean age of 13.1 years.	Hyrax expansion screw with or without mini-screws	None	Nasal airway dimensions (AR): - Minimum cross-sectional area; - Nasal cavity volume.	Immediate (post-expansion); 3 months (post-retention)	All three expanders (tooth-tissue-borne, tooth-borne, and bone-borne) significantly increased nasal volume and minimal cross-sectional area. Magnitude of nasal airway gains was comparable across appliance designs, with no meaningful between-group differences. Enlarged nasal dimensions were maintained at short-term follow-up. Appliance selection can prioritize other clinical factors since nasal airway outcomes were equivalent.
Mehta et al. (2022) [45]	USA	RCT	**Based on the RME appliance type:** 1. *MARPE Group:* 20 (gender not specified) with a mean age of 13.69 ± 1.74 years. 2. *RPE Group:* 21 (gender not specified) with a mean age of 13.9 ± 1.14 years.	MARPE Group: Mini-screw-assisted expander RPE Group: Conventional tooth-borne expander	19 Gender: not specified Mean age: 13.3 ± 1.49	Nasal airway morphology (CBCT): - Nasal cavity dimensions; - Nasal widths.	Time period T1 (post-expansion) to T2 (post-treatment): - MARPE group (2 years 7 months); - RPE group (2 years 9 months); - Control group (2 years 7 months).	Both MARPE and RPE effectively widened the nasal cavity, with MARPE showing superior long-term skeletal stability. In the short term, both appliances significantly increased the alar base width and the posterior and anterior nasal cavity widths compared with the controls. In the long term, MARPE demonstrated a sustained increase in posterior nasal cavity width, suggesting more stable skeletal expansion than RPE. MARPE provides a more stable and lasting skeletal expansion, making it a preferable option for long-term airway and structural enhancement.
Shetty et al. (2022) [73]	India	Prospective non-randomized clinical study	**Based on the treatment protocols for maxillary expansion:** 1. *RME Group:* 8 (4 M, 4 F) with a mean age of 12.5 ± 1.3 years. 2. *Alt-RAMEC (Alternate RME and Constriction) Group:* 7 (5 M, 2 F) with a mean age of 10.4 ± 1.8 years.	RME group: Hyrax-type expander Alt-RAMEC Group: Hyrax-type expander and Petit Face mask	None	Airway volume (CBCT): - Maxillary sinus; - Pharyngeal volume; - Hyoid bone position.	RME group: 5 months (post-retention); Alt-RAMEC group: 3 months (post-retention)	Both RME and Alt-RAMEC protocols significantly increase maxillary sinus volume, while producing no meaningful changes in pharyngeal airway dimensions or hyoid bone position. RME showed a greater effect than Alt-RAMEC, suggesting a stronger role in craniofacial development, although differences in pharyngeal airway volume and hyoid bone position were not statistically significant.
Caruso et al. (2023) [60]	Italy	Retrospective clinical study	14 M = 6 F = 8 Mean age: 8 ± 1	Hyrax-type rapid expander and Delaire mask	None	Upper airway dimensions (cephalometric linear measurements): - Nasopharynx; - Oropharynx; - Hypopharynx.	18 months: - RME phase for 12 months; - Delaire mask for 6 months; - 2 months after mask removal.	Combined therapy increases sagittal dimensions of the nasopharynx and oropharynx. Airway patency improves alongside OSAS-related clinical conditions. Protocol appears effective for Class III children with concomitant OSAS.
de Juliàn-Lòpez et al. (2023) [66]	Spain	Retrospective controlled clinical study	37 M = 19 F = 18 Mean age: not specified	Hyrax-type rapid expander	13 M = 5 F = 8 Age: not specified	Upper airway dimensions (CBCT)	RME group: ≈1.4 years; Control: ≈1.9 years	RME significantly increases upper airway volume in growing patients, producing a gain greater than expected from natural growth alone.
Gokce et al. (2023) [46]	Turkey	RCT	46 M = 16 F = 30 Mean age: not specified **Based on the RME appliance type:** 1. *TTB Group:* 15 (gender not specified) with a mean age of 12.5 years. 2. *TB Group:* 15 (gender not specified) with a mean age of 12.8 years. 3. *BB Group:* 16 (gender not specified) with a mean age of 13.1 years.	TTB Group: Acrylic-covered occlusal, palatal, and buccal tooth surfaces. TB Group: Hyrax screw soldered to bands on molars/premolars. BB Group: Mini-screw-assisted expander	None	Airway patency and breathing quality during sleep: - AHI; - Oxygen Desaturation Index (ODI); - Minimum oxygen saturation; - Supine AHI.	3 months (post-retention)	RME did not improve OSA severity (no meaningful change in AHI/ODI/minimum SpO_2_). No between-appliance differences in sleep outcomes were detected. All appliance types produced similar skeletal/dental maxillary expansion, but this did not translate into improvements in PSG. RME alone should not be relied upon as an OSA therapy; adjunctive or alternative management is recommended.
Korayem et al. (2023) [67]	Saudi Arabia	Retrospective controlled clinical study	52 Gender: not specified Mean age: not specified	Hyrax-type rapid expander	52 Gender: not specified Mean age: not specified	Upper airway dimensions (CBCT): - Volume; - Minimum cross-sectional area.	6 months (post-retention)	RME does not produce a significant additional increase in upper airway volume or MCA compared to natural growth in untreated controls. However, younger patients, with an earlier skeletal age at the onset of treatment, tended to show more favorable airway adaptations, suggesting that early intervention may have a greater positive impact.
Teixeira et al. (2023) [47]	Brazil	RCT	**Based on the RME appliance type:** 1. *Expander with Differential Opening (EDO) Group:* 24 (11 M, 13 F) with a mean age of 7.6 ± 0.9 years. 2. *Fan-Type Expander (FE) Group:* 24 (10 M, 14 F) with a mean age of 7.8 ± 0.9 years.	EDO Group: appliance with two screws: anterior and posterior regions of the palate. FE Group: appliance with one anterior screw with a posterior hinge.	None	Nasal cavity dimensions (CBCT): - Nasal cavity width; - Nasal cavity height.	6 months (post-retention)	Both expanders enlarged skeletal nasal cavity dimensions in growing patients. EDO achieved greater transverse widening in the lower third of the nasal cavity, both anterior and posterior. Middle and upper nasal widths increased similarly with EDO and FE. Nasal cavity height changes were comparable between devices. EDO may be preferred when prioritizing inferior nasal airway enlargement (e.g., oral breathing/OSA), while either device is suitable for general nasal widening.
Tsolakis et al. (2023) [22]	Greece	Prospective non-controlled clinical trial	14 M = 6 F = 8 Mean age: 10.82 ± 11.34	Hyrax-type rapid expander	None	Upper airway dimension (CBCT): - Volume; - Minimum cross-sectional area.	T1: Post-expansion (immediately after RPE) T2: 6 months post-expansion (retention)	RPE significantly increased both airway volume and MCA. Although airway volume slightly decreased after the six-month retention period, it remained higher than before treatment. The MCA showed a stable, sustained improvement, indicating that RPE effectively enhances and maintains nasal airway patency over time.
Bariani et al. (2024) [72]	Brazil	Prospective non-controlled clinical trial	24 M = 16 F = 8 Mean age: 10 ± 1.8 **Based on OAHI values:** *1. Primary Snoring group* (PS, OAHI value <1): 13 (9 M, 4 F) with a mean age of 10.3 ± 1.7 years; *2. Obstructive Sleep Apnea group* (OSA, OAHI value ≥1): 11 (7 M, 4 F) with a mean age of 9.7 ± 1.8 years.	Hyrax-type rapid expander	None	Respiratory function metrics: - PSG parameters. - Quality of life (QOL) questionnaires.	6 months (post-retention)	Treating SDB remains essential given symptoms/behavior linked to OAHI. RME is a plausible adjunct/alternative for children with persistent SDB and maxillary transverse deficiency. In the OSA subgroup, RME was associated with better quality of life and improvements in somatic complaints, aggressiveness, and visuospatial memory.
Ronsivalle et al. (2024) [61]	Italy	Retrospective cohort study	**Based on two age-defined treatment groups:** 1. *Early Expansion Group (EEG):* 25 (11 M, 14 F) with a mean age of 8.6 ± 1.1 years. 2. *Late Expansion Group (LEG):* 23 (10 M, 13 F) with a mean age of 12.2 ± 1.2 years.	Hyrax-type rapid expander	None	Upper airway dimensions (CBCT): - Nasal cavity volume; - Pharyngeal airway total volume; - Partial PA volumes; - Minimal cross-sectional area.	12 months (including 6 months retention)	At 12 months, both early (EEG) and late (LEG) groups showed significant increases in nasal cavity and total pharyngeal airway volumes. Nasopharynx, velopharynx, and oropharynx volumes all increased in both cohorts; CSmin and palatal width also rose. Increases were larger in younger patients, with a notably greater nasopharyngeal gain in the EEG. Deviation analysis suggests that part of the nasopharyngeal enlargement reflects reduced adenotonsillar tissue, more evident on the EEG.
Zreaqat et al. (2024) [37]	Malaysia	Prospective longitudinal study	47 M = 29 F = 18 Mean age: 10.29 ± 1.21	Hyrax-type rapid expander	None	Upper airway dimensions (CBCT): - Nasal cavity volume; - Nasopharynx volume; - MCA. Respiratory parameters (PSG).	6 months (post-retention)	RME is an effective therapeutic option for children with obstructive sleep apnea and maxillary constriction. The treatment produced significant improvements in upper airway dimensions, particularly in the nasal cavity and nasopharynx, along with notable enhancements in respiratory function. Increases in airway volume and reductions in respiratory disturbances suggest that RME can contribute to better sleep quality and breathing efficiency in pediatric patients.

**Table 4:** Evaluation of evidence certainty with the GRADE tool.

	OUTCOMES	
	Airway Morphology	Breathing Function
**Number of studies (participants)**	33 studies (1379 patients)	14 studies (509 patients)
**Study design**	Mainly observational study; six RCTs	Mainly observational study; four RCTs
**Risk of Bias**	Not serious ^a^	Not serious ^a^
**Inconsistency**	Not serious ^b^	Serious ^c^
**Indirectness**	Not serious ^d^	Serious ^e^
**Imprecision**	Not serious ^f^	Serious ^g^
**Publication of bias**	Not serious ^h^	Not serious ^h^
**Effect of direction**	Consistent improvement (≈88% of studies show enlargement; no study shows worsening)	Overall favorable (≈71% positive; ≈29% neutral/mixed; no study shows worsening)
**Overall GRADE** **certainty**	⨁⨁◯◯ Low	⨁◯◯◯ Very low

^a^ harmonized RoB2/ROBINS-I, weighted by sample; High/Critical < 20% and sensitivity unchanged. ^b^ consistent enlargement in nasal/nasopharyngeal segments; variability in oropharynx judged clinically explainable (age, follow-up, methods). ^c^ mixed populations and heterogeneous endpoints yielded discordant findings. Subgroup analyses did not fully explain heterogeneity. ^d^ pediatric cohorts; imaging endpoints directly aligned with the PICO; limited co-interventions; short-to-medium follow-up. ^e^ mixed pediatric populations and heterogeneous functional endpoints reduce the direct applicability of a single aggregated estimate. ^f^ large cumulative sample; multiple significant effects in nasal/nasopharyngeal segments; uncertainty in oropharynx/timing not decision-changing. ^g^ total sample split across endpoints; OIS not met for PSG; several wide or crossing CIs; short-term regressions in some measures. ^h^ not suspected/undetected based on qualitative assessment; formal tests not feasible due to endpoint heterogeneity.

## Data Availability

All data are contained within the article or Supplementary Materials.

## Supplementary Materials

The following supporting information can be downloaded at: https://www.mdpi.com/article/10.3390/jcm14248861/s1, Table S1: Characteristics of the included studies on rapid maxillary expansion with RPE; Table S2: Characteristics of included studies on rapid maxillary expansion comparing different devices.; Table S3: PRISMA 2020 Checklist. References [21,22,50,52,53,54,55,57,58,59,60,61,35,62,63,64,65,66,67,68,69,70,71,36,72,73,37,39,41,47,48,49], References [38,40,42,43,44,45,46,51,56] and Reference [76] are cited in Table S1, Table S2 and Table S3, respectively.
