# Supplementary material for: Relationship Between Maxillary Transverse Deficiency and Respiratory Problems: A Systematic Review of the Effectiveness of Devices over the Past Decade
Source: J Clin Med. 2025 Dec 15;14(24):8861. doi: 10.3390/jcm14248861 (PMC12733806; doi:10.3390/jcm14248861)
Supplement: Supplementary file 1 [file jcm-14-08861-s001.zip › Table_S1.pdf]

Table S1. Characteristics of the included studies on rapid maxillary expansion with RPE.

Study	Parameters used	Pre-treatment values (T0)	Post-treatment values at each time point (T1/T2)	Differences found	Main findings
Fastuca et al. (2015a)	- Airway volume<br>- Respiratory function	Airway volume (11358.02 ± 4391.2 mm^3)<br><br>PSG parameters:<br>- SpO2 (90.2 ± 1.3 %)<br>- AHI (5.01 ± 1.5)	Airway volume (15014.24 ± 7143.4 mm3)<br><br>PSG parameters:<br>- SpO2 (95.92 ± 1.5 %)<br>- AHI (1.45 ± 0.6)	Morphological Changes: airway volume increased by 3656 ± 5915.1 mm^3.<br><br>Functional Changes:<br>- Improvement in SpO2 by 5.72 ± 1.95 %;<br>- Reduction in AHI events by 3.56 ± 1.32 events.	The study found significant increases in total airway volume, SpO2, and AHI after RME treatment, with all parameters recorded at T1 being greater than those at T0 ($p<.05$).<br>No correlation was observed between changes in total airway volume and SpO2 or AHI.<br>The effect sizes for airway volume and AHI were statistically significant, ranging from 0.65 to 4.13.<br>Notably, the increases in SpO2 and AHI were clinically relevant, although total airway volume changes did not correlate with SpO2 and AHI changes.
Fastuca et al. (2015b)	- Airway volume<br>- Respiratory function	- Total Airway volume: 16.995 ± 5163 mm^3<br>- Upper airway volume: 7716 ± 3324 mm^3<br>-Middle airway volume: 5856 ± 2371 mm^3<br>- Lower airway volume: 3451 ± 1613 mm^3<br><br>PSG parameters:<br>- SpO2 (90.7 ± 5.1 %)<br>- AHI (5.8 ± 1.1)	- Total Airway volume: 22.389 ± 7610 mm^3<br>- Upper airway volume: 10.021 ± 3946 mm^3<br>-Middle airway volume: 7001 ± 2890 mm^3<br>- Lower airway volume: 5366 ± 2685 mm^3<br><br>PSG parameters:<br>- SpO2 (96.1 ± 4.5 %)<br>- AHI (1.6 ± 0.9)	Morphological Changes:<br>- Total airway volume increased by 5394 ± 2317 mm^3;<br>-Upper airway volume increased by 2305 ± 1014 mm^3;<br>- Middle airway volume increased by 1144 ± 835 mm^3;<br>- Lower airway volume increased by 1915 ± 662 mm^3.<br><br>Functional Changes:<br>- Improvement in SpO2 by 5.3 ± 0.6 %;	RME significantly increased upper, middle, and lower airway volumes ($p<0.05$).<br>Improvements in SpO2 and AHI were observed ($p<0.001$).<br>Baseline airway volumes correlated negatively with changes in oxygen saturation.<br>RME effectively improved respiratory function in patients with nasal obstruction.

				- Reduction in AHI events by 4.2 ± 1.5 events.	
Izuka et al. (2015)	1. Volumetric and cross-sectional changes in the upper airway:<br>- Transverse width of the anterior (ANF) and posterior (PNF) portion of the nasal floor;<br>- Airway volume of the nasopharynx and nasal cavities (VNN);<br>- Airway volume of the oropharynx (VO).<br><br>2. Quality of Life (QOL) Questionnaire	**Upper Airway Dimensions**<br>- ANF Width: 16.3 ± 1.7 mm<br>- PNF Width: 22.6 ± 2.5 mm<br>- VNN: 6114.4 ± 3490.4 mm³<br>- VO: 6378.2 ± 2357.5 mm³<br><br>**QOL Questionnaire**<br>Mean scores not reported	**Upper Airway Dimensions**<br>- ANF Width: 19.1 ± 1.8 mm<br>- PNF Width: 25.4 ± 3.0 mm<br>- VNN: 7760.5 ± 3841.4 mm³<br>- VO: 7828.8 ± 4109.9 mm³<br><br>**QOL Questionnaire**<br>Mean scores not reported	**Upper Airway Dimensional Changes**<br>- Both ANF and PNF showed a mean increase of 2.8 mm ($p < 0.001$).<br>- The VNN significantly increased by a mean of 1646.1 mm³ after RME ($p < 0.001$).<br>- While the VO also increased by a mean of 1450.6 mm³, this change was not statistically significant ($p = 0.066$).<br><br>**QOL Questionnaire**<br>***1. Physical Suffering***<br>The overall domain showed a highly significant improvement ($p < 0.001$).<br>***2. Sleep Disturbance***<br>This domain improved significantly ($p < 0.001$).<br>***3. Speech or Swallowing Problems***<br>The overall domain showed a p of 0.048, indicating borderline statistical significance.<br>***4. Emotional Distress***<br>The overall domain showed a significant improvement ($p = 0.006$).<br>***5. Activity Limitations***<br>The overall domain did not show a	RME is an effective treatment that not only creates significant dimensional changes in the upper airway, specifically in the nasal cavity and nasopharynx, but also markedly enhances the overall quality of life for patients suffering from mouth breathing and maxillary atresia.

				statistically significant improvement (p = 0.114).<br>***6. Parents' Concern about Snoring***<br>This domain showed a highly significant reduction in concern (p = 0.001).	
Cappellette et al. (2017a)	- Total nasomaxillary complex volume;<br>- Nasal cavity volume;<br>- Oropharynx volume.	**Total Volume**:<br>- RME group: 59.82 ± 11.5 mm^3<br>- Control group: 55.57 ± 8.1 mm^3<br>**Nasal Volume**:<br>- RME group: 33.42 ± 6.02 mm^3<br>- Control group: 34.43 ± 5.06 mm^3<br>**Oropharynx Volume**:<br>- RME group: 10.26 ± 2.42 mm^3<br>- Control group: 7.53 ± 1.53 mm^3	**Total Volume**:<br>- RME group: 69.32 ± 11.9 mm^3<br>- Control group: 55.75 ± 8.2 mm^3<br>**Nasal Volume**:<br>- RME group: 38.45 ± 6.33 mm^3<br>- Control group: 34.48 ± 5.08 mm^3<br>**Oropharynx Volume**:<br>- RME group: 12.95 ± 2.9 mm^3<br>- Control group: 7.57 ± 1.52 mm^3	**Overall Volumetric Increase**<br>The RME group showed a significant increase in the total volume of the nasomaxillary complex (p<0.001) compared to the control group.<br><br>**Specific Region Increases**<br>- Nasal Volume: The RME group experienced a mean nasal volume increase statistically significant (p < 0.001).<br>- Oropharynx Volume: A mean increase in the oropharynx volume of the RME group was statistically significant (p < 0.001).	The study highlights the significant positive effects of RME on the nasomaxillary complex, particularly in mouth-breathing patients with transverse maxillary deficiency.<br>RME was found to induce a volumetric expansion across the entire nasomaxillary complex.<br>This expansion was observed not only in the complex but also in its individual structures.<br>Specific structures that demonstrated volumetric expansion due to RME include the nasal cavity, the oropharynx, and the right and left maxillary sinuses.
Cappellette et al. (2017b)	- Nasal width;<br>- Nasal height;<br>- Nasal area (nasal width X nasal height).	**Nasal width**<br>M: 28.7 ± 2.8 mm<br>F: 28.5 ± 3.2 mm<br>**Nasal height**<br>M: 53.9 ± 3.4 mm<br>F: 51.6 ± 4.3 mm<br>**Nasal area**<br>M: 1551.8 ± 206.5 mm^2<br>F: 1473.1 ± 214.5 mm^2	**Nasal width**<br>M: 31.3 ± 3.27 mm<br>F: 31.9 ± 3.61 mm<br>**Nasal height**<br>M: 56.4 ± 3.65 mm<br>F: 54.2 ± 4.79 mm<br>**Nasal area**<br>M: 1770.7 ± 248.6 mm^2<br>F: 1730.8 ± 263.1 mm^2	**Sex-Specific Differences (T0)**<br>Females had a smaller nasal height compared to males (p = 0.025).<br>**Sex-Specific Differences (T1)**<br>Males showed significantly greater nasal height (p=0.05) compared to females.<br>**Sex-Specific Differences (T0-T1)**	Significant transverse gains with T0→T1 increases of height +2.5 mm, nasal width +2.9 mm, nasal area +235.4 mm^2, maxillary width +3.6 mm, intermolar width +6.5 mm<br>Broad skeletal and nasal expansion with larger nasal cavity volume.

				Significant changes in nasal width were observed in the female group (p = 0.027).	Sex differences were observed at baseline and post-treatment, yet overall transversal change remained comparable between sexes.
Fastuca et al. (2017)	- Nasal floor width;<br>- Nasal wall width.	**Nasal Floor Width**<br>- HX-6 group: 18.79 ± 2.36 mm<br>- HX-E group: 19.07 ± 2.71 mm<br>- HS-E group: 17.13 ± 4.18 mm<br><br>**Nasal Wall Width**<br>- HX-6 group: 26.01 ± 1.79 mm<br>- HX-E group: 29.17 ± 1.90 mm<br>- HS-E group: 27.80 ± 3.01 mm	**Nasal Floor Width**<br>- HX-6 group: 21.84 ± 3.12 mm<br>- HX-E group: 22.17 ± 3.02 mm<br>- HS-E group: 20.03 ± 3.97 mm<br><br>**Nasal Wall Width**<br>- HX-6 group: 28.37 ± 3.04 mm<br>- HX-E group: 31.62 ± 2.73 mm<br>- HS-E group: 30.47 ± 2.12 mm	**Within-Group Differences (T0 vs. T1)**<br>*Nasal Floor Width*<br>Significant increases were observed in all groups:<br>HX-6: p = 0.00004<br>HX-E: p = 0.00138<br>HS-E: p = 0.00478<br><br>*Nasal Wall Width*<br>Significant increases were shown across all groups:<br>HX-6: p = 0.00019<br>HX-E: p = 0.00017<br>HS-E: p = 0.00622<br><br>**Between-Group Differences**<br>No statistically significant differences were found among the three groups	The study investigates the effects of RME with different appliance designs and anchorage methods. RME effectively produces a significant skeletal transverse expansion of the nasal region in growing patients. The study found that no significant differences in nasal effects are expected whether the RME appliance is anchored onto deciduous teeth, with or without palatal acrylic coverage. This suggests that the choice between permanent or deciduous tooth anchorage, or the specific expander design, does not result in a statistically significant difference in nasal expansion.
Lo Giudice et al. (2017)	- Nasal cavity width (NW);<br>- Total nasal volume (TNV).	**RME group**<br>Nasal cavity width: 24.12 ± 1.13 mm<br>Total nasal volume: 17.84 ± 4.21 cm³<br><br>**SME group**<br>Nasal cavity width: 25.21 ± 2.68 mm<br>Total nasal volume: 15.81 ± 3.31 cm³	**RME group**<br>Nasal cavity width: 27.25 ± 1.97 mm<br>Total nasal volume: 24.14 ± 1.68 cm³<br><br>**SME group**<br>Nasal cavity width: 27.88 ± 3.30 mm<br>Total nasal volume: 17.06 ± 3.57 cm³	Both RME and SME increased skeletal (NW) and volumetric (TNV) nasal dimensions.<br><br>Absolute increases: RME > SME → NW +3.13 mm vs. +2.67 mm; TNV +1.68 cm³ vs. +1.25 cm³.<br><br>Percentage-based efficacy: SME	Manual segmentation and nasal volume computation were highly reliable, with small method errors (<0.3 mm linear; ~0.37 cm³ volumetric), suitable for assessing RME/SME effects and supporting diagnosis.<br>Percentage metrics PW% and NW%

				showed lower proportional skeletal effects (NW% 32.8%) compared to RME (NW% 46.1%).	better describe protocol efficacy than absolute PW and NW, accounting for differing expansion needs across patients.
Almuzian et al. (2018)	- Lower nasal cavity (LNC);<br>- Upper nasopharynx (UNP);<br>- Retropalatal (velo-pharyngeal) space, subdivided into the upper retropalatal (URP) and lower retropalatal (LRP) spaces.	LNC:<br>4785 ± 2195 mm³<br>UNP:<br>2736 ± 1395 mm³<br>URP space:<br>527 ± 827 mm³<br>LRP space:<br>3305 ± 2265 mm³	LNC:<br>5600 ± 3374 mm³<br>UNP:<br>3101 ± 1374 mm³<br>URP space:<br>492 ± 731 mm³<br>LRP space:<br>2994 ± 2226 mm³	**Nasopharyngeal Volumetric Changes:**<br>- The UNP space expanded significantly (P=0.04) in both genders, showing a similar response pattern.<br>- The LNC space gained further volume, with a 17% increase for the overall sample, although not statistically significant.<br>- The URP space was significantly reduced (p = 0.04).	RME significantly augmented the volume of the UNP. This expansion is attributed to anterior displacement of the palatal shelves, which pivot around the pterygoid-maxillary junction, thereby advancing the posterior nasal spine and increasing UNP volume. Conversely, the URP space was found to be significantly reduced, losing almost one-sixth of its original volume. A moderate correlation was also noted between changes in the UNP and URP spaces. The immediate expansion of the LNC and UNP secondary to RME may be associated with reduced nasal resistance and improved nasal breathing.
Badreddine et al. (2018)	Skeletal variables measured:<br>1. Nasal Height;<br>2. Height of the Pyriform Aperture;<br>3. Width of the Pyriform Aperture;<br>4. Total Length of the Nose.	**Skeletal Variables**<br>*Nasal Height*:<br>- RME group: 44.81 ± 4.01 mm<br>- Control group: 44.93 ± 4.35 mm<br>*Height of Pyriform Aperture*:<br>- RME group: 31.52 ± 3.42 mm<br>- Control group: 31.24 ± 3.51 mm<br>*Width of Pyriform Aperture*:	**Skeletal Variables**<br>*Nasal Height*:<br>- RME group: 46.32 ± 3.94 mm<br>- Control group: 45.05 ± 4.21 mm<br>*Height of Pyriform Aperture*:<br>- RME group: 32.78 ± 3.40 mm<br>- Control group: 31.14 ± 3.24 mm	**Skeletal Changes**<br>- The RME group showed statistically significant increases ($p < 0.05$) in all assessed skeletal variables. In contrast, the control group exhibited no significant alterations in these structures.<br>- The most pronounced skeletal	In the short term, RME resulted in significant changes in all nasal skeletal and soft tissue variables assessed three months after expansion.<br>It was found that nasal soft tissues accompanied skeletal changes in a nearly 1:1 ratio.

	Soft tissue variables measured:<br>1. Nasal Soft Tissue Height;<br>2. Nasal Soft Tissue Length;<br>3. Alar Width;<br>4. Width of the Soft Tissue Insertion.	- RME group: 21.64 ± 1.73 mm<br>- Control group: 21.28 ± 1.98 mm<br>*Total Length of the Nose*:<br>-RME group: 47.25 ± 3.35 mm<br>- Control group: 47.50 ± 3.09 mm<br><br>**Soft Tissue Variables**<br>*Width of the Soft Tissue Insertion*:<br>- RME group: 32.59 ± 2.84 mm<br>- Control group: 32.71 ± 1.72 mm<br>*Alar Width*:<br>- RME group: 32.68 ± 3.27 mm<br>- Control group: 32.69 ± 1.74 mm<br>*Height of the Nasal Soft Tissue*:<br>- RME group: 47.95 ± 4.07 mm<br>- Control group: 47.49 ± 4.69 mm<br>*Length of the Nasal Soft Tissue*:<br>- RME group: 15.89 ± 1.62 mm<br>- Control group: 15.41 ± 1.38 mm	*Width of Pyriform Aperture*:<br>- RME group: 23.62 ± 2.45 mm<br>Control group: 21.28 ± 1.88 mm<br>*Total Length of the Nose*:<br>- RME group: 47.78 ± 3.53 mm<br>- Control group: 47.71 ± 2.83 mm<br><br>**Soft Tissue Variables**<br>*Width of the Soft Tissue Insertion*:<br>- RME group: 34.02 ± 2.85 mm<br>- Control group: 32.78 ± 1.89 mm<br>*Alar Width*:<br>- RME group: 33.81 ± 3.32 mm<br>- Control group: 32.29 ± 2.08 mm<br>*Height of the Nasal Soft Tissue*:<br>- RME group: 49.74 ± 4.39 mm<br>- Control group: 47.69 ± 4.67 mm<br>*Length of the Nasal Soft Tissue*:<br>- RME group: 16.57 ± 1.67 mm<br>- Control group: 15.66 ± 1.57 mm	change was observed in the width of the pyriform aperture, which increased by +1.98 mm (+9.15%) in the RME group. This difference was highly significant when compared to the control group ($p < 0.001$).<br>- The height of the pyriform aperture also significantly increased by +1.26 mm (+4.00%) in the RME group.<br>- Significant increases were also noted in nasal height (+1.51 mm, +3.37%) and total nasal length (+0.53 mm, +1.12%) within the RME group.<br>**Soft Tissue Changes**<br>- All soft tissue variables measured in the RME group demonstrated significant increases ($p < 0.05$) between the pre- and post-RME time points, unlike the control group which showed no significant changes.<br>- The width of the soft tissue insertion showed the greatest increase among soft tissue variables in the EG, with an average increase of +1.43 mm (+4.39%). This was also the most significant difference when comparing the RME with the control group ($p = 0.002$).	Specifically, for every millimeter of skeletal increase, there was an approximately 0.95 mm (0.90%) increase in soft tissue.

				- The alar width increased by +1.13 mm (+3.46%) in the RME group.<br>- Increases were also observed in the height (+1.79 mm, +3.73%) and length (+0.68 mm, +4.28%) of the nasal soft tissue in the RME group.	
Di Vece et al. (2018)	- Upper nasal airway patency;<br>- Nasal airway resistance during inspiration and expiration.	Median nasopharynx obstruction score = 1.5 (IQR 1-3).<br><br>Inspiration: Mean nasal resistance (0.9 ± 0.55 Pa/cm³/s).<br>Expiration: Mean nasal resistance (0.85 ± 0.47 Pa/cm³/s).:	Median nasopharynx obstruction score = 1 (IQR 0-1).<br><br>Inspiration: Mean nasal resistance (0.46 ± 0.31 Pa/cm³/s).<br>Expiration: Mean nasal resistance (0.46 ± 0.29 Pa/cm³/s).	A significant decrease in nasopharynx obstruction after RPE was observed ($p<0.001$).<br><br>The quantitative reductions ranged from 0.23 to 0.66 Pa/cm³/s for inspiration and from 0.20 to 0.58 Pa/cm³/s for expiration, noted as significant at $p<0.001$.	Significant reduction in nasopharynx obstruction was observed after palatal expansion ($p<0.001$). Total nasal resistance significantly decreased for both inspiration and expiration after treatment ($p<0.001$). A positive correlation existed between changes in nasopharynx obstruction and expiration nasal airway resistance (rho = 0.38; p = 0.03).<br>Palatal expansion improved upper airway patency in patients with mild to moderate breathing difficulties.
Lotfi et al. (2018)	- Nasal cavity volume;<br>- Nasopharynx volume;<br>- Oropharynx volume;<br>- Hypopharynx volume.	**Hypopharynx volume**<br>*Group A:* 2253.97 ± 822.06 mm³<br>*Group B:* 1946.88 ± 532.45 mm³<br><br>**Nasal cavity volume**<br>*Group A:* 18666.7 ± 5221.6 mm³<br>*Group B*: 19487.40 ± 2744.50 mm³<br><br>**Nasopharynx volume**<br>*Group A:* 3673.92 ± 2593.9 mm³	**Hypopharynx volume**<br>*Group A:* 2232.24 ± 875.71 mm³<br>*Group B:* 1943.92 ± 528.80 mm³<br><br>**Nasal cavity volume**<br>*Group A:* 21372.2 ± 5700.3 mm³<br>*Group B*: 20542.32 ± 3351.23 mm³<br><br>**Nasopharynx volume**<br>*Group A:* 4130.15 ± 2401.93 mm³	**Within-Group**<br>*Nasal Cavity Volume*<br>- Group A showed a significant increase ($p < 0.0001$).<br>- Group B showed a significant increase ($p = 0.0010$).<br>*Nasopharynx Volume*<br>- Group A demonstrated a significant increase ($p = 0.0006$).<br>- Group B did not show a significant change.	The changes in airway volume following RME treatment are significantly influenced by the rate of expander activation.<br>A more rapid activation rate leads to greater increases in the volume of the nasal cavity and nasopharynx compared to a slower activation rate.

		Group B: 3443.91 ± 1384.24 mm³<br><br>**Oropharynx volume**<br>*Group A:* 10190.7 ± 4990.9 mm³<br>*Group B:* 11352.50 ± 2612.59 mm³	*Group B:* 3457.20 ± 1413.14 mm³<br><br>**Oropharynx volume**<br>*Group A:* 10286 ± 4929.22 mm³<br>*Group B:* 11424.01 ± 2955.01 mm³	**Between-Group**<br>*Hypopharynx Volume (T0)*<br>A significant difference was observed between Group A and Group B (p = 0.035)<br>*Nasal Cavity Volume (T1)*<br>Significant differences were detected between the two groups (p < 0.0001).<br>*Nasopharynx Volume (T1)*<br>The changes showed a significant difference between the groups (p = 0.0035).	The study found that the rate at which the expander is activated directly affects the magnitude of airway volume changes after RME.<br>This suggests a dose-dependent relationship where higher expansion rates yield more significant volumetric gains in these specific airway regions.
Ottaviano et al. (2018)	- Nasal flow and patency;<br>- Nasal resistance.	**PNIF**<br>*RME GROUP*<br>T0: 80 L/min<br>*CONTROL GROUP*<br>T0: 90 L/min<br><br>**AAR**<br>*RME GROUP*<br>T0: 0.1 Pa·s/ml<br>*CONTROL GROUP*<br>T0: 0.1 Pa·s/ml	**PNIF**<br>*RME GROUP*<br>T1: 110 L/min<br>T2: 120 L/min<br>*CONTROL GROUP*<br>T1: 90 L/min<br>T2: 90 L/min<br><br>**AAR**<br>*RME GROUP*<br>T1: 0.07 Pa·s/ml<br>T2: 0.08 Pa·s/ml<br>*CONTROL GROUP*<br>T1: 0.1 Pa·s/ml<br>T2: 0.1 Pa·s/ml	*RME GROUP:*<br>- PNIF showed significant improvement at T1 and T2 compared to T0 (p=0.003 and p=0.0002, respectively).<br>- AAR did not show significant changes across the three time intervals.<br><br>*CONTROL GROUP:*<br>No significant changes in any of the measured variables over the same periods.<br><br>*Comparison between groups in T0-T2:*<br>- PNIF values increased significantly in the RME group compared to the control (p = 0.0005).<br>- No significant changes were	The study found a significant increase in PNIF values after RME at T1 and T2 compared to the control group.<br>No significant improvement in nasal resistances was observed when measured by AAR after RME.<br>The study suggests that RME may enhance nasal airflow.

				observed in AAR values.	
Ribeiro et al. (2018)	- Nasal cavity geometry including minimum cross-sectional areas (MCA1, MCA2) and nasal space volumes (VOL1, VOL2);<br>- Nasal cavity patency.	**MCA1**<br>- Left Nasal Cavity: 0.25 cm²:<br>- Right Nasal Cavity: 0.30 cm².<br><br>**MCA2**<br>- Left Nasal Cavity: 0.36 cm²;<br>- Right Nasal Cavity: 0.32 cm².<br><br>**VOL1**<br>- 1.13 cm³ for right nasal cavity;<br>- 1.07 cm³ for left nasal cavity.<br><br>**VOL2**<br>- 2.43 cm³ for right nasal cavity;<br>- 2.32 cm³ for left nasal cavity.	**MCA1**<br>- Left nasal cavity: M2 = 0.40 cm²; M3 = 0.24 cm².<br>- Right nasal cavity: M2 = 0.35 cm²; M3 = 0.31 cm².<br><br>**MCA2**<br>- Left nasal cavity: M2 = 0.43 cm²; M3 = 0.42 cm².<br>- Right nasal cavity: M2 = 0.38 cm²; M3 = 0.35 cm².<br><br>**VOL1**<br>- Right nasal cavity: M2 = 1.22 cm³; M3 = 1.23 cm³.<br>- Left nasal cavity: M2 = 1.10 cm³; M3 = 1.09 cm³.<br><br>**VOL2**<br>- Right nasal cavity: M2 = 2.69 cm³; M3 = 2.36 cm³.<br>- Left nasal cavity: M2 = 2.70 cm³; M3 = 2.15 cm³.	The measurements for MCA1 did not exhibit statistically significant changes across the evaluation moments (M1, M2, and M3), despite slight variations between the left and right sides.<br><br>MCA2 demonstrated a statistically significant increase immediately post-RME (M2) compared to M1, but this gain tended to revert at M3, indicating a transient augmentation in the anterior nasal geometry.<br><br>VOL1 exhibited a statistically significant increase from M1 to M2 ($p=0.025$), with values remaining elevated at M3, albeit without clear statistical significance when compared to M2.<br><br>VOL2 demonstrated minimal variation across the evaluation periods, with slight increases observed but lacking statistically significant differences.	The study found that RME significantly increased the anterior region of the nasal cavity immediately after the RME treatment. However, there was a tendency for this increase to return to values close to the initial cross-sectional area after the retention period. No significant differences were observed between the right and left sides of the nasal cavity.

Abdalla et al. (2019)	- Pharyngeal airway volume;<br>- Minimal cross-sectional area (MCA).	Airway volume<br>*RME GROUP:* 12873.73 mm^3<br>*CONTROL GROUP*: 12216.05 mm^3<br><br>MCA<br>*RME GROUP:* 126.53 mm^2<br>*CONTROL GROUP*: 125.97 mm^2	**Airway volume**<br>*RME GROUP:* 17460.92 mm^3<br>*CONTROL GROUP:* 15794.43 mm^3<br><br>**MCA**<br>*RME GROUP:* 164.69 mm^2<br>*CONTROL GROUP:* 169.54 mm^2	Both groups showed a significant increase in the airway volume (RME group $p <.001$; control group $p = .006$) and MCA (RME group $p = .018$; control group $p = .020$) between T0 and T1<br><br>The difference between the groups regarding airway and MCA changes was not statistically significant	The RME group and the matched control group exhibited significant increases in upper airway volume and minimal cross-sectional area (MCA) over time. However, the increase in the RME group was not statistically significant compared to the control group. Tooth-borne RME did not lead to significant changes in upper airway dimensions compared to the control group.
Helal et al. (2019)	- 16-item questionnaire (sleep apnea symptoms, breathing patterns, sleep quality, fatigue, and behavior);<br>- Nasal cavity area.	**Sleep apnea & breathing**<br>- 37.2% snoring half the time;<br>- 22.4% always snoring;<br>- 27.4% snoring loudly;<br>- 62.2% heavy breathing;<br>- 15.5% trouble breathing;<br>- 10.2% stopping breathing;<br>- 63.5% oral breathing;<br>- 61.9% dry mouth;<br>- 10.2% apnea episodes.<br>Mean symptoms: 3.14 ± 1.93<br><br>**Sleep quality & fatigue**<br>- 37.1% unrefreshed sleep;<br>- 31.9% hard to wake;<br>- 24.4% daytime sleepiness.<br>Mean symptoms: 1.21 ± 1.45<br><br>**Behavioral issues**<br>- 42.7% fidgeting;<br>- 41.4% easily distracted;<br>- 35.3% interrupting;<br>- 27.8% difficulty organizing tasks.<br>Mean symptoms: 2.07 ± 2.16<br><br>**Nasal cavity**:<br>Mean 3.6 mm^2	**Sleep apnea & breathing**<br>- 7.0% snoring half the time;<br>- 3.6% loud snoring;<br>- 24.4% heavy breathing;<br>- 0% morning headache;<br>- 32.1% dry mouth;<br>- 0% apnea episodes.<br>Mean symptoms: 1.26 ± 1.21<br><br>**Sleep quality & fatigue**<br>- 12.4% unrefreshed sleep;<br>- 14.3% hard to wake;<br>- 8.9% daytime sleepiness.<br>Mean symptoms: 0.43 ± 0.84<br><br>**Behavioral issues**<br>- 38.2% fidgeting;<br>- 33.3% easily distracted;<br>- 25.9% interrupting;<br>- 16.7% difficulty organizing tasks.<br>Mean symptoms: 1.64 ± 2.01<br><br>**Nasal cavity**:<br>Mean 4.1 mm^2	After RME, significant reductions were observed in breathing and sleep apnea symptoms (mean 3.14 → 1.26, $p < 0.001$), including decreased snoring, reduced heavy breathing, decreased dry mouth, and fewer apnea episodes.<br><br>Sleep quality and fatigue improved markedly (mean 1.21 → 0.43, $p < 0.001$), with fewer reports of unrefreshed sleep and daytime sleepiness. Behavioral symptoms also decreased (mean 2.07 → 1.64, $p = 0.02$).<br><br>CBCT confirmed an increase in nasal cavity area (+4.1 mm^2).	RME produced clear benefits in children, with parents reporting improvements in behavior, reduced daytime fatigue, and better sleep quality. Breathing patterns also improved, with reductions in symptoms such as snoring and mouth breathing. Objective CBCT data confirmed an increase in nasal cavity area, supporting clinical perceptions.

Erdur et al. (2020)	- Pharyngeal airway (PA) volumes (upper, lower, and total);<br>- Maxillary sinus volume (MSV).	**PA Volume**<br>*ARME Group:*<br>- Upper: 7632.67 ± 2054.36 mm^3<br>- Lower: 17635.41 ± 5108.52 mm^3<br>- Total: 25268.08 ± 6753.78 mm^3<br>*RME Group:*<br>- Upper: 7237.16 ± 1835.39 mm^3<br>- Lower: 17453.28 ± 4304.47 mm^3<br>- Total: 24690.44 ± 6108.04 mm^3<br><br>**MSV**<br>*ARME Group:*<br>- Affected: 12425.52 ± 3218.03 mm^3<br>- Non-affected: 14376.81 ± 3891.55 mm^3<br>- Total: 26802.33 ± 6081.47 mm^3<br>*RME Group:*<br>- Right: 13168.73 ± 3032.83 mm^3<br>- Left: 12985.92 ± 4728.92 mm^3<br>- Total: 26154.65 ± 6089.57 mm^3	**PA Volume**<br>*ARME Group:*<br>- Upper: 9362.59 ± 2190.51 mm^3<br>- Lower: 18126.07 ± 4894.73 mm^3<br>- Total: 27488.66 ± 6836.57 mm^3<br>*RME Group:*<br>- Upper: 9580.39 ± 2730.73 mm^3<br>- Lower: 18014.26 ± 5284.35 mm^3<br>- Total: 27594.65 ± 6891.52 mm^3<br><br>**MSV**<br>*ARME Group:*<br>- Affected: 14603.46 ± 3973.58 mm^3<br>- Non-affected: 15024.63 ± 4079.49 mm^3<br>- Total: 29628.09 ± 6427.07 mm^3<br>*RME Group:*<br>- Right: 15392.51 ± 4047.62 mm^3<br>- Left: 14978.72 ± 4115.59 mm^3<br>- Total: 30371.23 ± 6581.73 mm^3	**PA Volume Differences**<br>*Upper:*<br>The RME group showed a significantly greater increase compared to the ARME group (p = .032).<br>*Lower:*<br>No significant intergroup difference was found.<br>*Total:*<br>Changes were significantly greater in the RME group, though this difference was not statistically significant.<br><br>**MSV Differences**<br>*Affected/Right MSV:*<br>The RME group showed a significantly greater increase compared to the affected side in the ARME group (p = .047).<br>*Non-affected/Left MSV:*<br>The RME group had a significantly greater increase compared to the non-affected side in the ARME group (p = .008).<br>*Total MSV:*<br>The changes were significantly greater in the RME group compared to the ARME group (p = .032)	RME was effective in increasing pharyngeal airway and maxillary sinus volumes in patients with bilateral maxillary deficiency. ARME was also effective, correcting a true unilateral posterior crossbite while contributing to increased airway and sinus volumes. Overall, both RME and ARME are valid options for treating maxillary deficiencies, with positive impacts on upper airway dimensions.

Lanteri et al. (2020)	- Nasal cavity volume (NCavV);<br>- Nasopharynx volume (NsPxV);<br>- Maxillary sinus volume (MSV).	**NCavV**<br>- SME group: 1271 ± 364 mm³<br>- RME group: 1216 ± 715 mm³<br><br>**NsPxV**<br>- SME group: 3663 ± 821 mm³<br>- RME group: 3568 ± 855 mm³<br><br>**MSVs**<br>- SME group: Right 8806 ± 1102 mm³, Left 8575 ± 983 mm³<br>- RME group, Right 8546 ± 713 mm³, Left 9063 ± 1101 mm³	**NCavV**<br>- SME group: 1701 ± 399 mmm³<br>- RME group: 1715 ± 518 mm³<br><br>**NsPxV**<br>- SME group: 5406 ± 821 mm³<br>- RME group: 5254 ± 812 mm³<br><br>**MSVs**<br>- SME group: Right 9358 ± 938 mm³, Left 9140 ± 1225 mm³<br>- RME group: Right 9314 ± 988 mm³, Left 9888 ± 1287 mm³	**Within-Group**<br>Significant increases were observed in NCavV, NsPxV, and MSVs after treatment with both SME and RME appliances.<br><br>**Between-Group**<br>When comparing the volumetric changes (ΔT1-T0), no statistically significant differences were found for any of the evaluated upper airway segments.	Both SME and RME are effective in treating maxillary hypoplasia in growing patients during the mixed dentition stage. Both approaches significantly increased pharyngeal airway and maxillary sinus volumes, and no statistically significant differences were found between SME and conventional Hyrax-RME appliances, indicating comparable orthopedic effects on the upper airway tract.
Abate et al. (2021)	- Forced Vital Capacity (FVC);<br>- Forced Expiratory Volume in the First Second (FEV1);<br>- Tiffenau Index (IT%);<br>- Forced Expiratory Flow at 25-75% of Vital Capacity (FEF 25-75%);<br>- Tidal Volume (TV).	**Oral Breathers**<br>- FVC: 4.25 ± 0.52<br>- FEV1: 3.33 ± 0.68<br>- IT%: 78.44 ± 6.18<br>- FEF 25-75%: 95.15 ± 6.06<br>- TV: 502.54 ± 59.90<br><br>**Nasal Breathers**<br>- FVC: 4.60 ± 0.63<br>- FEV1: 3.63 ± 0.63<br>- IT%: 78.95 ± 6.17<br>- FEF 25-75%: 98.95 ± 6.04<br>- TV: 538.96 ± 60.61	**Oral Breathers**<br>**T1**<br>- FVC: 4.65 ± 0.81<br>- FEV1: 3.66 ± 0.74<br>- IT%: 78.76 ± 6.78<br>- FEF 25-75%: 100.25 ± 8.34<br>- TV: 568.33 ± 61.76<br>**T2**<br>- FVC: 4.67 ± 0.82<br>- FEV1: 3.72 ± 0.71<br>- IT%: 79.71 ± 6.88<br>- FEF 25-75%: 102.03 ± 8.13<br>- TV: 587.50 ± 67.52<br><br>**Nasal Breathers**<br>**T1**<br>- FVC: 4.71 ± 0.77<br>- FEV1: 3.68 ± 0.67<br>- IT%: 77.83 ± 4.23<br>- FEF 25-75%: 102.33 ± 8.54<br>- TV: 579.34 ± 67.65<br>**T2**<br>- FVC: 4.68 ± 0.79<br>- FEV1: 3.74 ± 0.59<br>- IT%: 79.79 ± 3.97<br>- FEF 25-75%: 103.87 ± 7.44	**Within-Group**<br>*Oral Breathers:*<br>Significant overall improvements ($p < 0.001$) were observed in FVC, FEV1, IT%, and FEF 25–75%, with TV also improving ($p = 0.002$). For FVC and FEF 25–75%, the main improvement occurred from T0 to T1, with no further significant change from T1 to T2. FEV1 and IT% showed no significant change from T0 to T1 but improved significantly from T1 to T2. TV increased significantly from T0 to T1 and from T0 to T2, with no significant change between T1 and T2.<br><br>*Nasal Breathers:*<br>FVC ($p < 0.05$), FEF	RME led to measurable enhancements in respiratory performance for both oral and nasal breathers. Significant gains were observed in FEV1 and IT%, likely due to the reduction of elevated peripheral airway resistances present before treatment. In oral breathers, FVC, FEF 25–75%, and TV reached values comparable to those of nasal breathers after treatment, indicating a shift toward more physiological breathing patterns. Twelve months post-treatment, no significant differences were detected between

			- TV: 584.35 ± 57.04	25–75% (p < 0.001), and TV (p = 0.002) showed significant overall improvements. FVC and FEF 25–75% improved significantly from T0 to T1 and from T0 to T2, with no significant change between T1 and T2. TV followed the same pattern as in oral breathers. FEV1 and IT% showed no significant changes over time.<br><br>**Between-groups**<br>At T0, oral and nasal breathers differed significantly in FVC, FEF 25–75%, and TV (p < 0.05), but not in FEV1 or IT%. At T2, no significant differences were found in any spirometric parameter.	groups across spirometric indices, suggesting that the functional improvements were maintained over time.
Aljawad et al. (2021)	- Nasopharynx volume;<br>- Oropharynx volume;<br>- Minimal cross-sectional area.	**AIRWAY VOLUME**<br>*RME GROUP*<br>**- Nasopharynx:** 3123 ± 1310 mm^3<br>**- Retropalatal Segment of Oropharynx:** 4758 ± 1517 mm^3<br>**- Retroglossal Segment of Oropharynx**: 4800 ± 2028 mm^3<br>*CONTROL GROUP*<br>**- Nasopharynx:** 2782 ± 1479 mm^3<br>**- Retropalatal Segment of Oropharynx:** 4164 ± 2046 mm^3<br>**- Retroglossal Segment of Oropharynx**: 4141 ± 2287 mm^3<br><br>**MCA**<br>*RME GROUP*	**AIRWAY VOLUME**<br>*RME GROUP*<br>**- Nasopharynx:** 3782 ± 1690 mm^3<br>**- Retropalatal Segment of Oropharynx:** 5629 ± 1915 mm^3<br>**- Retroglossal Segment of Oropharynx**: 5788 ± 3296 mm^3<br>*CONTROL GROUP*<br>**- Nasopharynx:** 3094 ± 1130 mm^3<br>**- Retropalatal Segment of Oropharynx:** 4423 ± 1992 mm^3<br>**- Retroglossal Segment of Oropharynx**: 4088 ± 2536 mm^3<br><br>**MCA**<br>*RME GROUP*	**Within the RME group:**<br>- Significant increases were observed from before to after RME in nearly all measured parameters. This included nasopharyngeal (p = 0.018), retropalatal (p = 0.007), and retroglossal (p = 0.033) airway volumes.<br>- Retropalatal MCA significantly increased after RME treatment (p = 0.007).<br>- The retroglossal MCA change did not reach statistical	The study evaluated upper airway changes following RME in patients with maxillary transverse deficiency.<br>The findings indicate that the group treated with RME experienced significant increases in upper airway dimensions.<br>The MCA of the oropharynx showed a statistically significant increase in the RME group compared to the control group. This particular finding suggests a notable positive impact of

		**- Retropalatal Segment of Oropharynx**: 137.92 ± 45.14 mm²<br>**- Retroglossal Segment of Oropharynx**: 149.65 ± 48.51 mm²<br>*CONTROL GROUP*<br>**- Retropalatal Segment of Oropharynx:** 124.76 ± 78.41 mm²<br>**- Retroglossal Segment of Oropharynx**: 134.48 ± 74.0 mm²	**- Retropalatal Segment of Oropharynx**: 172.39 ± 69.75 mm²<br>**- Retroglossal Segment of Oropharynx**: 172.37 ± 68.87 mm²<br>*CONTROL GROUP*<br>**- Retropalatal Segment of Oropharynx:** 117.44 ± 75.63 mm²<br>**- Retroglossal Segment of Oropharynx**: 126.22 ± 71.27 mm²	significance ($p = 0.102$).<br>**Between the RME and control groups:**<br>- Increases in nasopharyngeal, retropalatal, and retroglossal airway volumes were greater in the RME group compared to the control group, but without significant differences.<br>- The MCA of the retropalatal airway showed a statistically significant increase in the RME group ($p = 0.038$).<br>- The difference in the MCA of the retroglossal airway between groups didn't show statistical significance ($p = 0.065$).	RME on this critical airway segment.
DiCosimo et al. (2021)	- Nasal cavity volumes (total, right, and left);<br>-Nasopharyngeal volume;<br>- Oropharyngeal volume;<br>- Minimum cross-sectional width (right and left).	***RME Group***<br>**Volumetric Measurements:**<br>- Total nasal cavity: 7971.6 ± 1801 mm³;<br>- Right nasal cavity: 4094.90 ± 1079.66 mm³;<br>- Left nasal cavity: 3813.10 ± 1138.28 mm³;<br>- Nasopharynx: 2815.88 ± 1037.34 mm³;<br>- Oropharynx: 7645.22 ± 2311.72 mm³.<br>**Minimum Cross-sectional Width Measurements:**<br>- Right nasal cavity: 0.34 ± 0.09 mm;<br>- Left nasal cavity: 0.33 ± 0.08 mm.<br><br>***Control Group***<br>**Volumetric Measurements:**<br>- Total nasal cavity: 7655.0 ± 2037.27 mm³;<br>- Right nasal cavity: 3954.0 ± 1375.53 mm³;	***RME Group***<br>**Volumetric Measurements:**<br>- Total nasal cavity: 10082.90 ± 2551.73 mm³;<br>- Right nasal cavity: 5063 ± 1323.3 mm³;<br>- Left nasal cavity: 4970.3 ± 1564.43 mm³;<br>- Nasopharynx: 3816.44 ± 1053.21 mm³;<br>- Oropharynx: 9994.40 ± 3511.89 mm³.<br>**Minimum Cross-sectional Width Measurements**:<br>- Right nasal cavity: 0.47 ± 0.12 mm;<br>- Left nasal cavity: 0.45 ± 0.11 mm.<br><br>***Control Group***<br>**Volumetric Measurements:**	***RME Group***<br>Total nasal cavity, right nasal cavity, nasopharynx, and oropharynx volumes increased post-RME (all $p<0.0001$); left nasal cavity $p=0.0006$.<br>Minimum cross-sectional width increased bilaterally (right +0.13±0.07 mm, left +0.11±0.06 mm, both $p<0.0001$).<br>Greater post-treatment increase on the left vs right nasal cavity ($p=0.045$).<br><br>***Control Group***<br>Oropharyngeal volume increased ($p=0.03$; +41.56%).	Significant post-RME volume gains in total nasal cavity (+30.82%), right (+26.53%), left (+38.82%), nasopharynx (+43.92%), and oropharynx (+33.76%).<br>The control group showed only an oropharyngeal increase.<br>Intergroup differences favored RME for total nasal cavity, right, left, and nasopharynx; no difference for oropharynx ($p=0.92$)<br>Minimum cross-sectional width increased bilaterally

		- Left nasal cavity: 3701.0 ± 1113.24 mm³;<br>- Nasopharynx: 2716.90 ± 11371.24 mm³;<br>- Oropharynx: 8307.0 ± 3383.73 mm³.<br>**Minimum Cross-sectional Width Measurements**:<br>- Right nasal cavity: 0.37 ± 0.11 mm;<br>- Left nasal cavity: 0.42 ± 0.15 mm.	- Total nasal cavity: 8027.34 ± 1807.87 mm³;<br>- Right nasal cavity: 4304.0 ± 1421.46 mm³;<br>- Left nasal cavity: 3723.39 ± 947.17 mm³;<br>- Nasopharynx: 2908.30 ± 1256.0 mm³;<br>- Oropharynx: 10551.0 ± 3680.72 mm³.<br>**Minimum Cross-sectional Width Measurements:**<br>- Right nasal cavity: 0.41 ± 0.13 mm;<br>- Left nasal cavity: 0.38 ± 0.14 mm.	Right nasal cavity volume change exceeded left (p=0.01).<br><br>***Between groups***<br>Larger volumetric increases with RME for total nasal cavity (p=0.002), right nasal cavity (p=0.04), left nasal cavity (p=0.01), and nasopharynx (p=0.004).<br>No intergroup difference for oropharyngeal volume change (p=0.92).<br>Greater increases in minimum cross-sectional width in the RME group for right (p=0.004) and left (p=0.0001) nasal cavities.	after RME and exceeded control. Overall pattern supports RME-driven nasal and nasopharyngeal enlargement beyond natural growth.
Feng et al. (2021a)	Volumes and cross-sectional areas of the nasopharynx, retropalatal, and retroglossal	**Cross-sectional areas**<br>- Nasopharyngeal: 320.16 ± 113.10 mm²<br>- Retropalatal: 193.54 ± 82.91 mm²<br>- Retroglossal: 237.96 ± 95.74 mm²<br><br>**Volumes**<br>- Nasopharyngeal: 3383.24 ± 1648.17 mm³<br>- Retropalatal: 5450.11 ± 1534.19 mm³<br>- Retroglossal: 4497.22 ± 2488.35 mm³<br><br>**AN ratio**<br>- Group 1: 0.49 ± 0.07 - Group 2: 0.72 ± 0.12	**Cross-sectional areas**<br>- Nasopharyngeal: 319.31 ± 115.78 mm²<br>- Retropalatal: 209.55 ± 89.96 mm²<br>- Retroglossal: 241.25 ± 107.32 mm²<br><br>**Volumes**<br>- Nasopharyngeal: 3769.95 ± 1670.48 mm³<br>- Retropalatal: 5781.52 ± 2188.29 mm³<br>- Retroglossal: 4590.56 ± 2161.19 mm³<br><br>**AN ratio**<br>- Group 1: 0.49 ± 0.15<br>- Group 2: 0.64 ± 0.16	**Changes in Cross-Sectional Areas and Volumes**<br>- After RME, the cross-sectional areas and volumes of airways tended to increase.<br>- The most pronounced volume increase was observed at the nasopharyngeal level, with a mean increase of 18.66%.<br>- Despite these increases, the changes in cross-sectional areas and volumes of the upper airway due to RME were not statistically significant.<br><br>**Changes in AN Ratio**	The study found that the effect of RME on volume changes in the nasopharyngeal airway could not be statistically verified. Despite this, there was an observed tendency for an increase in volume within the nasopharyngeal region.<br>The reduction of adenoid-associated nasal obstructions following RME also could not be statistically verified. However, a slight decrease in the AN ratio was noted, particularly in patients with adenoid hypertrophy.

				- Group 1: The mean AN ratio remained consistent at T0 and T1, with no significant difference.<br>- Group 2: The mean AN ratio in this group decreased from 0.72 at T0 to 0.64 at T1, representing an 11% reduction. However, this difference was also not statistically significant.	
Feng et al. (2021b)	Primary aerodynamic outcomes:<br>- Pressure Drop (ΔP);<br>- Maximum Midsagittal Velocity (Vms);<br>- Maximum Wall Shear Stress (Pws)	**ΔP**<br>insp. −4.00 ± 1.87 Pa<br>exp. 2.96 ± 2.56 Pa<br><br>**Vms**<br>insp. 2.48 ± 0.70 m/s<br>exp. 2.79 ± 1.09 m/s<br><br>**Pws**<br>insp. 1.29 ± 1.24 Pa<br>exp. 1.63 ± 1.85 Pa	**ΔP**<br>insp. −4.36 ± 2.45 Pa<br>exp. 2.81 ± 2.43 Pa<br><br>**Vms**<br>insp. 2.43 ± 0.92 m/s<br>exp. 2.28 ± 0.82 m/s<br><br>**Pws**<br>insp. 1.03 ± 1.32 Pa<br>exp. 0.93 ± 0.71 Pa	No statistically significant changes in ΔP or Pws after RME.<br><br>Vms at expiration decreased (2.79 → 2.28 m/s), close to significance (p=0.057).<br><br>Group differences: patients with higher AN ratio (≥0.6) showed persistently higher ΔP and Vms, but lower Pws, compared to AN ratio <0.6.	RME did not produce statistically significant changes in upper airway aerodynamic characteristics. Airflow dynamics remained largely unchanged after treatment.
Niu et al. (2021)	- Nasal Cavity (NC) Volume;<br>- Pharyngeal Airway (PA) Total Volume;<br>- Partial PA Volumes (nasopharynx, velopharynx, oropharynx);<br>- Minimal Cross-Sectional Area (Minimal CS):<br>- Minimal Hydraulic Diameter (Minimal DH).	**NC volume**<br>*RME group:* 10.795 ± 2.119 mm^3<br>*Control group*: 12.047 ± 2.518 mm^3<br><br>**PA volumes**<br>**1. Total PA:**<br>*RME group:* 8.263 ± 2.441 mm^3<br>*Control group*: 8.317 ± 3.180 mm^3<br>**2. Nasopharynx volume:**<br>*RME group:* 1.300 ± 665 mm^3<br>*Control group*: 1.665 ± 937 mm^3<br>**3. Velopharynx volume:**	**NC volume**<br>*RME group:* 12.467 ± 2.401 mm^3<br>*Control group*: 12.620 ± 2.239 mm^3<br><br>**PA volumes**<br>**1. Total PA:**<br>*RME group:* 10.192 ± 3.241 mm^3<br>*Control group*: 10.103 ± 3.452 mm^3<br>**2. Nasopharynx volume:**<br>*RME group:* 1.754 ± 789 mm^3<br>*Control group*: 1.975 ± 840 mm^3<br>**3. Velopharynx volume:**	**NC volume**<br>- Significantly lower in the RME group than controls at baseline (p = .031).<br>- Significant increase after treatment within the RME group (p < .001).<br>- Significant net increase compared to controls (p = .029).<br><br>**Total PA**<br>- No baseline difference between groups (p = .939).<br>- Significant increase within the RME	RME produced a measurable expansion of the nasal cavity, with particularly marked gains when palatal width increased by more than 2 mm. Although RME induced changes within the pharyngeal airway, total PA volume did not differ significantly from the control group. Using a validated evaluation method, the study found that

		RME group: 2.685 ± 1.086 mm^3<br>*Control group*: 2.710 ± 1.408 mm^3<br>**4. Oropharynx volume:**<br>*RME group:* 4.028 ± 1.670 mm^3<br>*Control group*: 3.937 ± 1.838 mm^3<br><br>**PA Cross-Sections**<br>**1. Minimal CS:**<br>*RME group:* 84.78 ± 36.94 mm^2<br>*Control group*: 102.14 ± 56.64 mm^2<br>**2. Minimal DH:**<br>*RME group:* 7.57 ± 1.95 mm<br>*Control group*: 8.13 ± 2.24 mm	*RME group:* 3.318 ± 1.487 mm^3<br>*Control group*: 3.396 ± 1.367 mm^3<br>**4. Oropharynx volume:**<br>*RME group:* 5.026 ± 2.255 mm^3<br>*Control group*: 4.825 ± 2.503 mm^3<br><br>**PA Cross-Sections**<br>**1. Minimal CS:**<br>*RME group:* 111.07 ± 56.24 mm^2<br>*Control group*: 116.63 ± 59.22 mm^2<br>**2. Minimal DH:**<br>*RME group:* 8.58 ± 2.48 mm<br>*Control group*: 8.84 ± 2.50 mm	group after treatment ($p < .001$).<br>- No significant net difference compared to controls ($p = .860$).<br><br>**Partial PA Volumes**<br>- No baseline differences for any subregion (all $p > .05$).<br>- Significant within-group increases in all subregions for the RME group: nasopharynx ($p < .001$), velopharynx ($p = .001$), oropharynx ($p = .013$).<br>- No significant net differences versus controls (all $p > .3$).<br><br>**Minimal CS**<br>- No baseline difference ($p = .136$).<br>- Significant increase within the RME group ($p = .002$).<br>- No significant net difference compared to controls ($p = .416$).<br><br>**Minimal DH**<br>- No baseline difference ($p = .268$).<br>- Significant increase within the RME group ($p = .001$).<br>- No significant net difference compared to controls ($p = .550$).	initially lower minimal cross-sectional area and minimal hydraulic diameter values in the RME group normalized after treatment.
Shetty et al. (2022)	- Maxillary Sinus Volume;<br>- Pharyngeal Airway Volume (nasopharynx, oropharynx, and hypopharynx);<br>- Hyoid Bone Position (H-	**RME Group**<br>*Maxillary Sinus Dimensions:*<br>- Right sinus volume: 6532.42 ± 1039.19 mm^3<br>- Left sinus volume: 6490.28 ± 1042.02 mm^3<br><br>*Pharyngeal Airway Volumes:*	**RME Group**<br>*Maxillary Sinus Dimensions:*<br>- Right sinus volume: 7052.74 ± 755.09 mm^3<br>- Left sinus volume: 6970.87 ± 680.43 mm^3<br>*Pharyngeal Airway Volumes:*	**Maxillary Sinus Volume Changes**<br>*RME Group:*<br>- A significant increase was observed in the total maxillary sinus volume, with a mean difference of 520 ±	RME significantly increases maxillary sinus volume.<br>No meaningful change in naso-, oro-, or hypopharyngeal dimensions or in hyoid position after RME.

	C3Rgn vertical distance, H-Rgn horizontal distance).	- Nasopharynx volume: 5426.75 ± 1329.4 mm³<br>- Oropharynx volume: 26240.63 ± 7111.21 mm³<br>- Hypopharynx volume was 3167.75 ± 1367.07 mm³<br>*Hyoid Bone Position:*<br>- H-C3Rgn: 7.15 ± 3.4 mm<br>- H-Rgn: 33.79 ± 6.82 mm<br><br>**Alt-RAMEC Group**<br>*Maxillary Sinus Dimensions:*<br>- Right sinus volume: 4490.99 ± 1237.25 mm³<br>- Left sinus volume: 4536.69 ± 1258.68 mm³<br>*Pharyngeal Airway Volumes:*<br>- Nasopharynx volume: 4907.29 ± 1073.06 mm³<br>- Oropharynx volume: 25446.14 ± 5678.25 mm³<br>- Hypopharynx volume: 3032.29 ± 817.31 mm³<br>*Hyoid Bone Position:*<br>- H-C3Rgn: 4.44 ± 1.87 mm<br>- H-Rgn: 31.98 ± 2.92 mm	- Nasopharynx volume: 5848.88 ± 1676.72 mm³<br>- Oropharynx volume: 26822.13 ± 5919 mm³<br>- Hypopharynx volume: 3341.5 ± 1926.98 mm³<br>*Hyoid Bone Position:*<br>- H-C3Rgn: 7.23 ± 3.85 mm<br>- H-Rgn: 34.8 ± 5.83 mm<br><br>**Alt-RAMEC Group**<br>*Maxillary Sinus Dimensions:*<br>- Right sinus volume: 5192.86 ± 1198.36 mm³<br>- Left sinus volume: 5216.01 ± 1234.27 mm³<br>*Pharyngeal Airway Volumes:*<br>- Nasopharynx volume: 5749 ± 1245.61 mm³<br>- Oropharynx volume: 26950 ± 5031.56 mm³<br>- Hypopharynx volume: 3780.29 ± 824.7 mm³<br>*Hyoid Bone Position*:<br>- H-C3Rgn: 3.88 ± 1.88 mm<br>- H-Rgn: 32.95 ± 2.12 mm	576.57 mm³ (p=0.038).<br>- The right maxillary sinus craniocaudal height also showed a significant increase (p=0.011).<br>*Alt-RAMEC Group*:<br>- A significant increase was observed in the right maxillary sinus craniocaudal height (p=0.006) and anteroposterior dimension (p=0.021), leading to an overall increase in the right maxillary sinus total volume (p=0.018). - The left maxillary sinus total volume increased significantly (p=0.017).<br>*Inter-group Comparison*:<br>- The RME group showed a statistically significantly greater increase in maxillary sinus volume ($p < 0.05$).<br><br>**Pharyngeal Airway Volume Changes**<br>*RME Group:*<br>- No statistically significant changes were observed in the pharyngeal airway volumes.<br>*Alt-RAMEC Group:*<br>- A statistically significant increase was found in the nasopharyngeal volume (p=0.017).<br>*Inter-group Comparison:*<br>- The increase in pharyngeal airway volume was generally greater in	AltRAMEC (with facemask) also increases maxillary sinus volume without altering pharyngeal airway segments or hyoid position.<br>Between protocols, RME yields a greater maxillary sinus volume gain than AltRAMEC.<br>Pharyngeal airway volume tends to increase more with RME, but the between-group difference is not statistically significant.<br>Both groups show forward hyoid posture trends that are not statistically significant.

				the RME group, but this difference was not statistically significant.<br>- The oropharynx volume was higher in the Alt-RAMEC group, but this difference was not statistically significant.<br><br>**Hyoid Bone Position Changes**<br>*RME Group:*<br>- No statistically significant changes were noted.<br>*Alt-RAMEC Group:*<br>- No statistically significant changes were noted.<br>*Inter-group Comparison*:<br>- The RME group showed a greater difference in hyoid bone position, but this was statistically insignificant.	
Caruso et al. (2023)	- Nasopharynx;<br>- Oropharynx;<br>- Hypopharynx..	**Rinopharynx**:<br>7.5 mm (IQR 6–11)<br><br>**Oropharynx**:<br>15.2 mm (IQR 14.3–18)<br><br>**Hypopharynx**:<br>10.2 mm (IQR 8–14)	**Rinopharynx**:<br>9.5 mm (IQR 8.4–14)<br><br>**Oropharynx**:<br>16.65 mm (IQR 15–19)<br><br>**Hypopharynx**:<br>13 mm (IQR 10–16)	The nasopharyngeal width increased from a median of 7.5 mm to 9.5 mm ($p = 0.009$).<br>The oropharyngeal dimension also showed an increase, rising from 15.2 mm to 16.65 mm ($p = 0.020$).<br>The hypopharyngeal dimension increased slightly from 10.2 mm to 13 mm, but this change was not statistically significant ($p = 0.095$).	Treatment with RME and the Delaire mask resulted in significant increases in nasopharyngeal and oropharyngeal dimensions, thereby improving upper airway patency in children with Class III malocclusion and OSAS.
de Juliàn-Lòpez et al. (2023)	Airway volume	*RME group*<br>14.1263 ± 4.3998 mm³<br><br>*Control group*<br>10.9113 ± 1.2496 mm³	*RME group*<br>18.0641 ± 4.5659 mm³<br><br>*Control group*<br>13.1689 ± 1.7897 mm³	RME group showed significantly greater airway volume increase than	Palatal disjunction increases upper airway volume beyond natural growth.

				controls (p=0.036; +31.8% vs. +20.9%). Baseline difference present at T0 (p=0.013), more pronounced at T1 (p<0.001). ~1,353 mm³ gain directly attributable to RME beyond natural growth.	Net treatment-attributable gain ≈ +1.35 cm³ over growth expectations. Effect does not depend on sex, age, facial profile, or growth pattern.
Korayem et al. (2023)	- Upper airway volume;<br>- Minimum cross-sectional area (MCA).	**Upper airway volume**<br>Control group: 12,227.12 mm³<br>RME group: 12,884.84 mm³<br><br>**MCA**<br>Control group: 126.04 mm²<br>RME group: 126.53 mm²	Airway volume<br>Control group: 15,805.54 mm³<br>RME group: 17,471.08 mm³<br><br>MCA<br>Control group: 170.61 mm²<br>RME group: 164.69 mm²	Airway volume increased significantly in both groups (control: p = 0.007; RME: p = 0.002), and MCA also showed significant growth (control: p = 0.041; RME: p = 0.002). However, when comparing the two groups, the RME effect was not statistically significant (p > 0.05).	Tooth-borne RME did not yield meaningful increases in upper-airway volume or minimum cross-sectional area compared with controls. More favorable airway modifications were observed in children with younger skeletal age at treatment start. Early timing may optimize modest airway benefits, but effects remain limited.
Teixeira et al. (2023)	- Nasal Cavity Width (lower, middle, and upper thirds);<br>- Nasal Cavity Height (lower, middle, and upper thirds).	**Anterior Region**<br>Upper width:<br>- EDO = 10.32 ± 2.83 mm<br>- FE = 10.75 ± 1.84 mm<br>Middle width:<br>- EDO = 21.11 ± 1.78 mm<br>- FE = 19.73 ± 1.52 mm<br>Lower width:<br>- EDO = 22.02 ± 1.69 mm<br>- FE = 21.38 ± 1.31 mm<br>Height:<br>- EDO = 31.42 ± 2.39 mm<br>- FE = 31.51 ± 1.99 mm<br><br>**Posterior Region**<br>Upper width:<br>- EDO = 3.61 ± 0.80 mm<br>- FE = 4.16 ± 0.92 mm<br>Middle width:<br>- EDO = 20.18 ± 2.61 mm<br>- FE = 21.11 ± 2.32 mm<br>Lower width:<br>- EDO = 26.45 ± 2.20 mm<br>FE = 25.98 ± 1.82 mm	**Anterior Region**<br>Upper width:<br>- EDO = 1.53 ± 1.55 mm<br>- FE = 1.19 ± 0.87 mm<br>Middle width:<br>- EDO = 2.19 ± 1.08 mm<br>- FE = 1.63 ± 0.69 mm<br>Lower width:<br>- EDO = 2.76 ± 0.89 mm<br>- FE = 2.10 ± 0.74 mm<br>Height:<br>- EDO = 1.04 ± 1.55 mm<br>- FE = 0.72 ± 0.76 mm<br><br>**Posterior Region**<br>Upper width:<br>- EDO = 0.33 ± 0.46 mm<br>- FE = 0.13 ± 0.20 mm<br>Middle width:<br>- EDO = 1.02 ± 0.72 mm<br>- FE = 0.62 ± 0.58 mm<br>Lower width:<br>- EDO = 2.33 ± 0.76 mm<br>FE = 1.22 ± 0.54 mm	Anterior region: the mean increase was 2.76 mm with the EDO compared to 2.10 mm with the FE (p = 0.007). Posterior region: the EDO achieved a mean increase of 2.33 mm versus 1.22 mm for the FE (p < 0.001). No significant intergroup differences were found in the middle or upper thirds of the nasal cavity. Similarly, changes in nasal cavity height, both anteriorly and posteriorly, were not statistically different	Both EDO and FE effectively increased the skeletal dimensions of the nasal cavity. The EDO produced a significantly greater transverse increase in the lower third of the nasal cavity compared to the FE, both in the anterior and posterior regions of the maxilla. This suggests that the EDO exerts a stronger orthopedic effect in the lower nasal area — the region most relevant to airway function. While both devices successfully expanded the nasal

		Height:<br>- EDO = 31.74 ± 3.83 mm<br>FE = 31.93 ± 2.66 mm	Height:<br>- EDO = 1.26 ± 1.55 mm<br>FE = 1.49 ± 1.13 mm	between the two expanders.	cavity, the EDO achieved a more substantial widening in the lower nasal region, making it potentially more advantageous for improving airflow in patients with oral breathing patterns or airway obstruction.
Tsolakis et al. (2023)	- Upper airway volume;<br>- Minimum cross-sectional area (MCA).	**Upper airway volume**<br>9292.2 ± 3595.0 mm³<br><br>**MCA**<br>5.0 ± 2.3 mm²	**Upper airway volume**<br>T1: 12,319.1 ± 3274.6 mm³<br>T2: 11,731.3 ± 3260.6 mm³<br><br>**MCA**<br>T1: 11.0 ± 4.5 mm²<br>T2: 10.3 ± 4.2 mm²	Airway volume increased markedly from T0 to T1 ($p < 0.001$) and remained significantly higher at T2 ($p = 0.001$). However, a slight decrease occurred between T1 and T2 ($p < 0.001$).<br><br>The MCA showed a significant increase at T1 ($p = 0.003$) and remained stable at T2 ($p = 0.056$).	RPE produces a significant immediate increase in nasal airway volume.<br>Volume remains above baseline after retention, with partial regression from the post-expansion peak.<br>Minimal cross-sectional area increases significantly post-expansion and is maintained through retention.<br>Overall effect is a durable enlargement of nasal passage caliber, with stable MCA and sustained, though slightly reduced, volume gains.
Bariani et al. (2024)	PSG parameters (AHI, SpO_2).<br><br>Quality of Life (QOL) Questionnaires:<br>- Obstructive Pediatric Sleep Questionnaire (PSQ);<br>- Obstructive Sleep Apnea 18-Item Quality-of-Life Questionnaire (OSA-18).	*PS group*<br>PSG parameters:<br>- Basal SpO2 (96.71 ± 0.87 %)<br>- Mean SpO2 (96.00 ± 1.18 %)<br>- Minimum SpO2 (92.54 ± 1.81 %)<br>- AHI (0.83 ± 1.08)<br><br>*OSA group*<br>PSG parameters:<br>- Basal SpO2 (96.11 ± 0.96 %)<br>- Mean SpO2 (95.12 ± 1.22 %)<br>- Minimum SpO2 (88.82 ± 2.99 %)	*PS group*<br>PSG parameters:<br>- Basal SpO2 (96.82 ± 0.89 %)<br>- Mean SpO2 (95.57 ± 1.66 %)<br>- Minimum SpO2 (92.15 ± 1.82 %)<br>- AHI (1.87 ± 3.65)<br><br>*OSA group*<br>PSG parameters:<br>- Basal SpO2 (95.60 ± 1.62 %)<br>- Mean SpO2 (94.54 ± 1.57 %)<br>- Minimum SpO2 (89.50 ± 3.10 %)	AHI shows a significant decrease in the OSA group after RME ($p = 0.011$).<br><br>Significant changes in Basal SpO2 for the PS group post-RME ($p=0.021$).<br><br>The minimum SpO2% showed a significant improvement in the OSA group ($p = 0.035$).	RME has a positive impact on the quality of life (QOL) for children with SDB, regardless of the severity of OAHI.<br>The quality of life improved significantly after RME, as indicated by OSA-18 scores.<br>RME may be an alternative treatment for children with SDB refractory to adenotonsillectomy, improving behavioral

		- AHI (2.26 ± 1.19)<br><br>*PSQ for all samples*<br>- Total Score 14.1;<br>- Snoring 6.7;<br>- Sleepiness 2.9;<br>- Behavior 4.4.<br><br>*OSA-18 for all samples*<br>- Sleep disturbance 20.0;<br>- Physical symptoms 18.2;<br>- Emotional symptoms 12.7;<br>- Daytime function 13.4;<br>- Caregiver concern 19.3.	- AHI (2.33 ± 1.41)<br><br>*PSQ for all samples*<br>- Total Score 5.4;<br>- Snoring 1.7;<br>- Sleepiness 1.6;<br>- Behavior 2.1.<br><br>*OSA-18 for all samples*<br>- Sleep disturbance 7.3;<br>- Physical symptoms 8.1;<br>- Emotional symptoms 5.6;<br>- Daytime function 7.6;<br>- Caregiver concern 7.2.	Significant reduction in the PSQ and total OSA-18 scores after RME treatment ($p < 0.01$).	and cognitive outcomes.
Ronsivalle et al. (2024)	- Nasal Cavity (NC) Volume;<br>- Pharyngeal Airway (PA) Total Volume;<br>- Partial PA Volumes (nasopharynx, velopharynx, oropharynx);<br>- Minimal Cross-Sectional Area (Minimal CS).	**NC volume**<br>*EEG group:* 8730.38 ± 1552.09 mm^3<br>*LEG group*: 14266.39 ± 2156.94 mm^3<br><br>**PA volumes**<br>**1. Total PA:**<br>*EEG group:* 5315.15 ± 1079.55 mm^3<br>*LEG group*: 9219.28 ± 2348.23 mm^3<br>**2. Nasopharynx volume:**<br>*EEG group:* 1655.13 ± 891.13 mm^3<br>*LEG group*: 3067.49 ± 1322.89 mm^3<br>**3. Velopharynx volume:**<br>*EEG group:* 1394.01 ± 479.30 mm^3<br>*LEG group*: 2705.31 ± 1190.95 mm^3<br>**4. Oropharynx volume:**<br>*EEG group:* 2266.01 ± 718.89 mm^3<br>*LEG group*: 3446.49 ± 1387.38 mm^3<br><br>**PA Minimal CS:**<br>*EEG group:* 82.78 ± 28.43 mm^2<br>*LEG group*: 93.48 ± 34.32 mm^2	**NC volume**<br>*EEG group:* 2524.76 ± 1233.47 mm^3<br>*LEG group*: 2673.32 ± 969.51 mm^3<br><br>**PA volumes**<br>**1. Total PA:**<br>*EEG group:* 2449.80 ± 498.83 mm^3<br>*LEG group*: 2167.11 ± 414.10 mm^3<br>**2. Nasopharynx volume:**<br>*EEG group:* 1045.37 ± 333.53 mm^3<br>*LEG group*: 738.17 ± 272.94 mm^3<br>**3. Velopharynx volume:**<br>*EEG group:* 490.80 ± 126.32 mm^3<br>*LEG group*: 531.10 ± 203.77 mm^3<br>**4. Oropharynx volume:**<br>*EEG group:* 913.63 ± 236.84 mm^3<br>*LEG group*: 897.85 ± 294.93 mm^3<br><br>**PA Minimal CS:**<br>*EEG group:* 39.21 ± 10.21 mm^2<br>*LEG group*: 30.14 ± 18.31 mm^2	A statistically significant difference was observed in the change of nasopharynx volume between the two groups ($p < 0.05$).<br><br>The change in minimal cross-sectional area showed a significant difference between the groups ($p < 0.05$).<br><br>No significant differences were found in the changes of the nasal cavity, total pharyngeal airway, velopharynx, and oropharynx volumes.	The study investigated the medium-term effects of RME on NC and PA volumes, considering age as a factor.<br>The main findings highlight significant volumetric increases in both early and late expansion groups, with some differences being more pronounced in younger patients, and emphasize that observed changes are not solely due to RME treatment.
Zreaqat et al. (2024)	Nasomaxillary Complex (CBCT):<br>- Nasal cavity volume;	**CBCT Measurements**<br>Nasal cavity volume: 16,350 ± 4287 mm^3<br>Nasopharynx volume: 3973 ± 1621 mm^3<br>Nasopharynx MCA: 84.72 ± 15.31 mm^2	**CBCT Measurements**<br>Nasal cavity volume: 18,789 ± 4770 mm^3<br>Nasopharynx volume: 4856 ± 1896 mm^3<br>Nasopharynx MCA: 116.46 ± 23.58 mm^2	The nasal cavity volume increased by an average of 2439 mm^3 ($p < .001$), the nasopharynx showed a significant enlargement of 883	Nasal cavity volume rose markedly (~+2.4 cm^3); nasopharyngeal volume and MCA increased; inter-sinus distance widened, while sinus volumes

- Nasopharynx volume and MCA.<br><br>Respiratory and Sleep Parameters (PSG):<br>- AHI;<br>- ODI;<br>- Sleep efficiency;<br>- Arousal index;<br>- Lowest SpO_2;<br>- Average SpO_2;<br>- Total sleep time;<br>- REM sleep.	**PSG Parameters**<br>Total sleep time: 7.83 ± 1.40 h<br>Sleep efficiency: 88.63 ± 0.76 %<br>AHI: 8.12 ± 3.89 events/h<br>ODI: 12.78 ± 5.12 events/h<br>Lowest SpO_2: 88.78 ± 4.65 %<br>Average SpO_2: 94.73 ± 2.75 %<br>Arousal index: 17.15 ± 4.97 events/h	**PSG Parameters**<br>Total sleep time: 7.90 ± 1.36 h<br>Sleep efficiency: 91.74 ± 0.83 %<br>AHI: 1.62 ± 1.32 events/h<br>ODI: 7.02 ± 2.37 events/h<br>Lowest SpO_2: 94.40 ± 4.55 %<br>Average SpO_2: 94.89 ± 2.69 %<br>Arousal index: 9.46 ± 2.78 events/h	mm^3 ($p = .008$), while the minimum cross-sectional area of the nasopharynx expanded by 31.74 mm^2 ($p < .001$).<br><br>In terms of respiratory function, the AHI decreased by 6.50 events/h ($p < .001$).<br>The ODI was reduced by 5.76 events/h ($p = .024$), and the lowest SpO_2 improved by 5.62% ($p < .001$).<br>Additionally, the arousal index dropped significantly by 7.69 events/h ($p < .001$), and sleep efficiency increased by 3.39% ($p = .035$).	did not change significantly.<br>AHI fell substantially (~−5–6 events/h /h) with several children normalizing (AHI<1); ODI decreased; lowest SpO_2 increased (~+5–6%); arousal index declined, and sleep efficiency improved.<br>RME enlarged upper-airway compartments and produced clinically meaningful improvements in PSG metrics in this OSA cohort.
