# Supplementary material for: Relationship Between Maxillary Transverse Deficiency and Respiratory Problems: A Systematic Review of the Effectiveness of Devices over the Past Decade
Source: J Clin Med. 2025 Dec 15;14(24):8861. doi: 10.3390/jcm14248861 (PMC12733806; doi:10.3390/jcm14248861)
Supplement: Supplementary file 1 [file jcm-14-08861-s001.zip › Table_S2.pdf]

**Table S2.** Characteristics of included studies on rapid maxillary expansion comparing different devices.

| Study | Parameters used | Pre-treatment values (T0) | Post-treatment values at each time point (T1/T2) | Differences found | Main findings |
|---|---|---|---|---|---|
| Kabalan et al. (2015) | Minimum cross-sectional area (MCA1, MCA2) and airway volume (VOL1, VOL2) changes | **TB group**<br>*VOL1*:<br>-0.15 ± 1.64 cm$^3$ (right);<br>0.19 ± 1.69 cm$^3$ (left).<br>*VOL2*:<br>1.10 ± 4.53 cm$^3$ (right);<br>-0.26 ± 1.77 cm$^3$ (left).<br>*MCA1:*<br>-0.02 ± 0.15 cm$^2$ (right);<br>0.03 ± 0.10 cm$^2$ (left).<br>*MCA2:*<br>0.03 ± 0.08 cm$^2$ (right);<br>1.02 ± 4.44 cm$^2$ (left).<br><br>**BB group**<br>*VOL1*:<br>0.03 ± 1.27 cm$^3$ (right);<br>-0.24 ± 1.35 cm$^3$ (left).<br>*VOL2*:<br>0.06 ± 1.36 cm$^3$ (right);<br>0.04 ± 1.26 cm$^3$ (left).<br>*MCA1:*<br>-0.01 ± 0.09 cm$^2$ (right);<br>-0.01 ± 0.11 cm$^2$ (left).<br>*MCA2:*<br>-0.03 ± 0.08 cm$^2$ (right);<br>-0.01 ± 0.09 cm$^2$ (left).<br><br>**Control group**<br>*VOL1*:<br>-1.15 ± 2.24 cm$^3$ (right);<br>0.05 ± 0.54 cm$^3$ (left).<br>*VOL2*:<br>-1.46 ± 2.15 cm$^3$ (right);<br>-0.10 ± 1.30 cm$^3$ (left).<br>*MCA1:*<br>1.30 ± 4.10 cm$^2$ (right);<br>0.03 ± 0.02 cm$^2$ (left).<br>*MCA2:*<br>-0.10 ± 0.13 cm$^2$ (right);<br>0.01 ± 0.08 cm$^2$ (left). | **TB group**<br>*VOL1*:<br>-0.19 ± 1.89 cm$^3$ (right);<br>0.07 ± 1.75 cm$^3$ (left).<br>*VOL2*:<br>0.13 ± 1.57 cm$^3$ (right);<br>-0.07 ± 1.76 cm$^3$ (left).<br>*MCA1:*<br>-0.04 ± 0.13 cm$^2$ (right);<br>0.03 ± 0.07 cm$^2$ (left).<br>*MCA2:*<br>0.03 ± 0.10 cm$^2$ (right);<br>-0.03 ± 0.12 cm$^2$ (left).<br><br>**BB group**<br>*VOL1*:<br>0.38 ± 1.48 cm$^3$ (right);<br>0.06 ± 1.36 cm$^3$ (left).<br>*VOL2*:<br>-0.23 ± 1.35 cm$^3$ (right);<br>0.38 ± 1.48 cm$^3$ (left).<br>*MCA1:*<br>-0.01 ± 0.12 cm$^2$ (right);<br>-0.01 ± 0.12 cm$^2$ (left).<br>*MCA2:*<br>-0.03 ± 0.08 cm$^2$ (right);<br>-0.02 ± 0.12 cm$^2$ (left).<br><br>**Control group**<br>*VOL1*:<br>-0.04 ± 1.21 cm$^3$ (right);<br>-0.10 ± 0.59 cm$^3$ (left).<br>*VOL2*:<br>0.05 ± 0.54 cm$^3$ (right);<br>-0.09 ± 1.21 cm$^3$ (left).<br>*MCA1:*<br>0.01 ± 0.05 cm$^2$ (right);<br>-0.02 ± 0.07 cm$^2$ (left).<br>*MCA2:*<br>0.03 ± 0.02 cm$^2$ (right);<br>0.01 ± 0.05 cm$^2$ (left). | Variability Across Groups:<br>data variability was high across all groups, showing no consistent pattern or predictable trend in airway response.<br><br>Group Comparisons: ANOVA tests revealed no statistically significant differences ($p > 0.05$) in nasal airway volume or MCA between the three groups.<br><br>Functional Parameters:<br>Changes in nasal airway volume and MCA from T0 to T1 were minor, inconsistent, and sometimes negative, with no clear difference between treated and control subjects. | RME, whether tooth-borne or bone-borne, does not produce significant or predictable improvements in nasal airway dimensions or function.<br>Across groups, no significant intergroup differences were detected (ANOVA $p>0.05$), and functional–morphologic correlations (AR vs CBCT) were largely absent; even the few positive findings were asymmetric between nostrils.<br>Outcomes were unpredictable, indicating RME should not be relied upon to enhance nasal airway function. |
| Motro et al. (2016) | Airway volume changes:<br>1. Total Airway Volume;<br>2. Nasopharynx Volume; | Not reported | Not reported | **Overall Airway Volume Expansion**<br>The total airway volume showed a highly significant increase from T0 to T1 ($p < 0.001$). | RME leads to significant expansion of both the nasopharynx and the oropharynx.<br>RME does not have any significant effect |

| | | | | | |
|---|---|---|---|---|---|
| | 3. Oropharynx Volume;<br>4. Laryngopharynx Volume. | | | **Segment-Specific Airway Expansion**<br>Both the nasopharynx and oropharynx demonstrated highly significant expansion ($p < 0.000$).<br>In contrast, the airway at the laryngopharynx did not change significantly ($p = 0.779$). | on the laryngopharyngeal airway.<br>The positive rhinological effects were comparable across all groups, regardless of the different RME appliances used. |
| Barzagani et al. (2018) | - Nasal airflow;<br>- Nasal airway resistance. | **Nasal Airflow**<br>*TB Group*: 181.0 ± 49.6 cm$^3$/s<br>*TBB Group*: 188.5 ± 54.7 cm$^3$/s<br><br>**Nasal Airway Resistance**<br>*TB Group*: 0.94 ± 0.38 Pa·s/cm$^3$<br>*TBB Group*: 0.88 ± 0.36 Pa·s/cm$^3$ | **Nasal Airflow**<br>*TB Group*: 186.2 ± 60.3 cm$^3$/s<br>*TBB Group*: 243.9 ± 91.4 cm$^3$/s<br><br>**Nasal Airway Resistance**<br>*TB Group*: 0.89 ± 0.32 Pa·s/cm$^3$<br>*TBB Group*: 0.66 ± 0.22 Pa·s/cm$^3$ | Nasal airflow: The TBB group showed a clear improvement compared to the TB group. The mean post-expansion airflow was higher by approximately 51.0 cm$^3$/s in the complete case analysis ($p = 0.018$)<br>Nasal resistance: The TBB group exhibited a significant reduction compared to the TB group. The mean difference was −0.21 Pa·s/cm$^3$, with a p-value of 0.016. | The TBB appliance was more effective than TB RME in improving nasal function. Patients treated with TBB RME showed significantly higher nasal airflow and lower nasal resistance compared to those treated with TB RME.<br>These improvements suggest that TBB RME provides a more efficient enhancement of nasal breathing. |
| Kavand et al. (2019) | - Nasal cavity volume;<br>- Nasopharynx volume;<br>- Oropharynx volume;<br>- Maxillary sinus volume. | **Nasal cavity volume**<br>*TB group*: 14860 ± 3109 mm$^3$<br>*BB group*: 15241 ± 3959 mm$^3$<br><br>**Nasopharynx volume**<br>*TB group:* 3760 ± 1630 mm$^3$<br>*BB group*: 3530 ± 1616 mm$^3$<br><br>**Oropharynx volume**<br>*TB group*: 11746 ± 4269 mm$^3$<br>*BB group:* 11045 ± 2186 mm$^3$ | **Nasal cavity volume**<br>*TB group*: 16726 ± 3041 mm$^3$<br>*BB group*: 17690 ± 3993 mm$^3$<br><br>**Nasopharynx volume**<br>*TB group:* 4580 ± 1819 mm$^3$<br>*BB group*: 4237 ± 1739 mm$^3$<br><br>**Oropharynx volume**<br>*TB group*: 12297 ± 3660 mm$^3$<br>*BB group:* 11329 ± 2642 mm$^3$ | **Airway Volume Changes**<br>*Nasal Cavity Volume*<br>While both groups showed significant increases individually ($p < .05$), the difference in the change ($T_2$-$T_1$) between the groups was not statistically significant.<br><br>*Nasopharynx Volume*<br>Both groups exhibited significant increases ($p < .05$), | Both tooth-borne and bone-borne rapid maxillary expanders significantly increased the volume of the nasal cavity and nasopharynx. Additionally, both methods led to an expansion of maxillary dental and skeletal widths.<br>Despite the individual increases in nasal cavity and nasopharynx volume, there was no |

| | | | | | |
|---|---|---|---|---|---|
| | | **Right maxillary sinus volume**<br>*TB group:* 13004 ± 3926 mm³<br>*BB group:* 13406 ± 4399 mm³<br><br>**Left maxillary sinus volume**<br>*TB group:* 12369 ± 4039 mm³<br>*BB group:* 13539 ± 4449 mm³ | **Right maxillary sinus volume**<br>*TB group:* 13739 ± 3759 mm³<br>*BB group:* 13695 ± 4090 mm³<br><br>**Left maxillary sinus volume**<br>*TB group:* 13184 ± 3821 mm³<br>*BB group:* 14242 ± 4125 mm³ | but the difference in the change between the two groups was not statistically significant.<br><br>*Oropharynx Volume*<br>There was no significant increase within either group. Consequently, there was no significant difference in the change between the two groups.<br><br>*Maxillary Sinus Volumes*<br>Neither group showed a significant increase in right or left maxillary sinus volume individually. The differences in changes between the two groups were also not statistically significant. | statistically significant difference in the magnitude of these airway volume changes when comparing the tooth-borne and bone-borne expansion groups. |
| Cheung et al. (2021) | Primary outcome: overall upper airway volume.<br><br>Secondary outcomes:<br>- Nasal cavity volume;<br>- Nasopharynx volume;<br>- Oropharynx volume;<br>- Hypopharynx volume;<br>- Functional airway volume. | **Nasal cavity**<br>Hyrax: 25,298.2 ± 9,403.8 mm³<br>Hybrid-Hyrax: 26,630.8 ± 5,659.0 mm³<br>Keles: 23,449.2 ± 7,856.0 mm³<br><br>**Nasopharynx**<br>Hyrax: 4,663.5 ± 2,691.4 mm³<br>Hybrid-Hyrax: 5,416.8 ± 2,194.0 mm³<br>Keles: 5,585.2 ± 3,073.1 mm³<br><br>**Oropharynx**<br>Hyrax: 9,465.6 ± 2,757.6 mm³<br>Hybrid-Hyrax: 11,651.8 ± 6,208.3 mm³<br>Keles: 10,517.4 ± 3,537.2 mm³<br><br>**Hypopharynx** | **Nasal cavity**<br>Hyrax: 26,364.3 ± 8,645.1 mm³<br>Hybrid-Hyrax: 29,319.5 ± 5,536.7 mm³<br>Keles: 24,725.6 ± 6,472.9 mm³<br><br>**Nasopharynx**<br>Hyrax: 4,637.4 ± 2,725.5 mm³<br>Hybrid-Hyrax: 6,362.4 ± 2,443.8 mm³<br>Keles: 6,764.5 ± 3,240.8 mm³<br><br>**Oropharynx**<br>Hyrax: 10,788.7 ± 4,047.3 mm³<br>Hybrid-Hyrax: 12,702.7 ± 5,678.1 mm³<br>Keles: 12,060.5 ± 1,973.9 mm³<br><br>**Hypopharynx** | **Total airway volume**<br>Small increases in all groups:<br>- Hybrid-Hyrax: +5902.1 mm³ (+8.3%);<br>- Keles: +3001.4 mm³ (+4.5%);<br>- Hyrax: +2537.9 mm³ (+3.8%).<br>No statistically significant overall differences between groups (p > 0.05).<br><br>**Functional airway volume**<br>No significant group differences (p = 0.26).<br><br>**Compartment-specific findings**<br>- Nasopharynx: Keles > Hyrax | RME produced modest increases in total upper airway volume and compartments (3.8–8.3%).<br>No significant differences were found among Hyrax, Hybrid-Hyrax, and Keles expanders, except for greater gains in the nasopharynx with Keles. |

| | | | | | |
|---|---|---|---|---|---|
| | | Hyrax: 3,494.6 ± 1,052.4 mm³<br>Hybrid-Hyrax: 3,441.9 ± 1,430.0 mm³<br>Keles: 3,273.1 ± 1,301.7 mm³<br><br>**Total airway volume**<br>Hyrax: 42,921.9 ± 12,744.1 mm³<br>Hybrid-Hyrax: 47,141.4 ± 11,544.2 mm³<br>Keles: 42,824.9 ± 11,054.4 mm³ | Hyrax: 3,289.1 ± 1,201.2 mm³<br>Hybrid-Hyrax: 3,451.3 ± 1,290.9 mm³<br>Keles: 2,924.5 ± 1,112.6 mm³<br><br>**Total airway volume**<br>Hyrax: 45,079.5 ± 12,087.1 mm³<br><br>Hybrid-Hyrax: 51,835.9 ± 10,598.0 mm³<br>Keles: 46,475.2 ± 9,539.3 mm³ | (statistically significant, p = 0.04).<br>- Nasal cavity, oropharynx, hypopharynx: No significant differences among expanders. | |
| Garib et al. (2021) | Nasal cavity width | **HH group**<br>28.81 ± 2.35 mm<br><br>**CH group**<br>28.33 ± 2.18 mm | **HH group**<br>2.26 ± 1.17 mm<br><br>**CH group**<br>1.11 ± 0.95 mm | Statistically significant differences were found in nasal cavity width expansion between the HH and CH groups (p = 0.004). | The HH expander was more effective than the CH in increasing nasal cavity width. The HH group showed significantly greater expansion, indicating a stronger orthopedic effect. |
| Gokce et al. (2022) | Minimum cross-sectional area (MCA1, MCA2, cm$^2$) and nasal cavity volume (VOL1, VOL2, cm³) changes by AR | **MCA1**<br>*TTB group:* 0.77 cm²<br>*TB group*: 0.77 cm²<br>*BB group*: 0.88 cm²<br><br>**MCA2**<br>*TTB group:* 0.94 cm²<br>*TB group*: 0.91 cm²<br>*BB group*: 0.99 cm²<br><br>**VOL**<br>*TTB group:* 5.87 cm³<br>*TB group*: 5.25 cm³<br>*BB group*: 5.83 cm³ | **MCA1**<br>*TTB group*<br>T1: 0.91 cm²<br>T2: 0.99 cm²<br>*TB group*<br>T1: 1.01 cm²<br>T2: 0.95 cm²<br>*BB group*<br>T1: 1.02 cm²<br>T2: 1.00 cm²<br><br>**MCA2**<br>*TTB group*<br>T1: 1.12 cm²<br>T2: 1.22 cm²<br>*TB group*<br>T1: 1.30 cm²<br>T2: 1.35 cm²<br>*BB group*<br>T1: 1.28 cm²<br>T2: 1.47 cm²<br><br>**VOL**<br>*TTB group*<br>T1: 6.40 cm³<br>T2: 7.03 cm³<br>*TB group*<br>T1: 6.75 cm³<br>T2: 7.11 cm³ | MCA1 Changes: While all groups showed significant increases in MCA1 from T0 to T1 and/or T2, inter-group comparisons revealed no statistically significant differences in these changes across the three appliance types.<br><br>MCA2 Changes: All groups experienced significant increases in MCA2 post-treatment. However, there was no significant difference in the magnitude of these changes between the groups at any time interval (T1-T0, T2-T0, T2-T1). | Tooth tissue-borne, tooth-borne, and bone-borne rapid maxillary appliances were all effective in statistically significantly increasing nasal volume and the minimal cross-sectional areas (MCA) after RME treatment.<br>The increases observed in nasal volume and MCA were similar across all groups, indicating that the specific appliance design had no significant effect on the nasal airway dimensions.<br>The increase in nasal cavity dimensions achieved through RME was maintained during short-term follow-up. |

| | | | | | |
|---|---|---|---|---|---|
| | | | *BB group*<br>T1: 7.02 cm³<br>T2: 8.35 cm³ | Nasal Volume Changes:<br>Although all groups showed significant increases in nasal volume, and the BB group specifically showed a statistically significant increase across all measured periods (T0-T1, T0-T2, T1-T2), the inter-group comparison for overall volume changes did not show a significant difference. | |
| Mehta et al. (2022) | - Nasal Cavity Dimensions: nasal height (NH), nasal length (NL), nasion-ANS height (NAH), ANS-PNS length (APL), and pyriform height (PH).<br>- Nasal Widths: alar width (AW), alar base width (ABW), anterior nasal cavity width (ANCW), and posterior nasal cavity width (PNCW). | **MARPE group**<br>NH = 50.79 ± 4.79 mm;<br>NL = 17.79 ± 1.88 mm;<br>NAH = 49.88 ± 5.36 mm;<br>APL = 51.10 ± 4.03 mm;<br>AW = 34.60 ± 2.88 mm;<br>ABW = 32.83 ± 2.76 mm;<br>PH = 39.03 ± 5.57 mm;<br>PNCW = 29.22 ± 3.46 mm;<br>ANCW = 20.02 ± 2.75 mm.<br><br>**RPE group**<br>NH = 51.12 ± 3.49 mm;<br>NL = 18.80 ± 2.43 mm;<br>NAH = 50.46 ± 3.35 mm;<br>APL = 51.96 ± 4.36 mm;<br>AW = 35.40 ± 3.03 mm;<br>ABW = 33.13 ± 3.11 mm;<br>PH = 38.85 ± 3.36 mm;<br>PNCW = 29.40 ± 2.76 mm;<br>ANCW = 19.66 ± 2.38 mm.<br><br>**Control group**<br>NH = 49.26 ± 3.93 mm;<br>NL = 17.24 ± 2.62 mm;<br>NAH = 50.01 ± 3.91 mm;<br>APL = 51.70 ± 4.42 mm;<br>AW = 33.42 ± 3.66 mm;<br>ABW = 31.82 ± 3.64 mm;<br>PH = 38.82 ± 3.50 mm;<br>PNCW = 28.92 ± 2.37 mm;<br>ANCW = 20.53 ± 2.36 mm. | **MARPE group**<br>NH = 52.75 ± 4.41 mm;<br>NL = 18.82 ± 2.49 mm;<br>NAH = 50.53 ± 4.27 mm;<br>APL = 52.53 ± 3.54 mm;<br>AW = 34.86 ± 2.54 mm;<br>ABW = 32.85 ± 2.23 mm;<br>PH = 39.50 ± 3.99 mm;<br>PNCW = 30.82 ± 3.14 mm;<br>ANCW = 20.71 ± 1.86 mm.<br><br>**RPE group**<br>NH = 54.02 ± 4.50 mm;<br>NL = 19.77 ± 2.74 mm;<br>NAH = 51.75 ± 2.61 mm;<br>APL = 52.64 ± 4.38 mm;<br>AW = 35.81 ± 3.08 mm;<br>ABW = 33.48 ± 2.79 mm;<br>PH = 40.18 ± 3.05 mm;<br>PNCW = 30.37 ± 3.33 mm;<br>ANCW = 20.65 ± 2.62 mm.<br><br>**Control group**<br>NH = 51.95 ± 4.43 mm;<br>NL = 18.49 ± 1.90 mm;<br>NAH = 51.26 ± 3.45 mm;<br>APL = 52.76 ± 4.35 mm;<br>AW = 33.84 ± 3.43 mm;<br>ABW = 32.38 ± 3.49 mm;<br>PH = 40.39 ± 4.11 mm;<br>PNCW = 29.48 ± 2.48 mm; | **Short-term changes (T0–T1)**<br>Both MARPE and RPE produced significant increases in nasal and maxillary widths, including ABW, PNCW, and ANCW ($p < .05$).<br>**Long-term changes (T0–T2)**<br>Both expansion groups showed lasting increases in NH, NL, NAH, APL, and PH ($p < .05$).<br>PNCW remained significantly higher in the MARPE group compared to RPE and controls at long-term follow-up ($p < .05$).<br>No significant differences were found in ANCW among groups. | Both MARPE and RPE produce short-term increases in alar base, anterior/posterior nasal cavity widths, and maxillary intercanine/intermolar widths.<br>Both reduce nasal septal deviation versus controls in the short term.<br>Long term, MARPE shows a sustained increase in posterior nasal cavity width; RPE does not.<br>Both maintain a small long-term decrease in septal deviation.<br>No adverse long-term change in soft-tissue nasal width. |

| | | | | | |
|---|---|---|---|---|---|
| | | | ANCW = 20.94 ± 2.77 mm. | | |
| Gokce et al. (2023) | Polygraphic Sleep Parameters:<br>- AHI: events/hour;<br>- Oxygen Desaturation Index (ODI): events/hour;<br>- Minimum Oxygen Saturation (%);<br>- Supine AHI: apnea-hypopnea index during supine sleep. | **AHI**<br>TTB group: 9.2<br>TB group: 5.4<br>BB group: 5.8<br><br>**ODI**<br>TTB group: 5.2<br>TB group: 6.2<br>BB group: 5.7<br><br>**Minimum Oxygen Saturation**<br>TTB group: 82%<br>TB group: 81%<br>BB group: 81%<br><br>**Supine AHI**<br>TTB group: 8<br>TB group: 7<br>BB group: 4.5 | **AHI**<br>TTB group: 8.0<br>TB group: 5.5<br>BB group: 5.1<br><br>**ODI**<br>TTB group: 5.6<br>TB group: 4.9<br>BB group: 4.2<br><br>**Minimum Oxygen Saturation**<br>TTB group: 80 %<br>TB group: 84 %<br>BB group: 82 %<br><br>**Supine AHI**<br>TTB group: 7<br>TB group: 3<br>BB group: 5 | AHI: Changes observed from baseline to follow-up were not statistically significant ($p = 0.631$).<br><br>ODI: Altered slightly in all groups, but without significant intergroup differences ($p = 0.858$).<br><br>Minimum Oxygen Saturation: No significant change within or between groups ($p = 0.534$).<br><br>Supine AHI: No significant variation from baseline or between groups ($p = 0.872$). | RME did not significantly improve the severity of obstructive sleep apnea.<br>However, all appliance types —tissue-borne, tooth-borne, and bone-borne —produced comparable maxillary expansion with similar effects. |
